# Neodymium as Metal Cofactor for Biological Methanol Oxidation: Structure and Kinetics of an XoxF1-Type Methanol Dehydrogenase

**DOI:** 10.1128/mBio.01708-21

**Published:** 2021-09-21

**Authors:** Rob A. Schmitz, Nunzia Picone, Helena Singer, Andreas Dietl, Kerstin-Anikó Seifert, Arjan Pol, Mike S. M. Jetten, Thomas R. M. Barends, Lena J. Daumann, Huub J. M. Op den Camp

**Affiliations:** a Department of Microbiology, Radboud University, Nijmegen, The Netherlands; b Department Chemie, Ludwig-Maximilians-Universität München, Munich, Germany; c Department of Biomolecular Mechanisms, Max Planck Institute for Medical Research, Heidelberg, Germany; University of California, Irvine

**Keywords:** *Methylacidimicrobium*, PQQ, lanthanides, methanol dehydrogenase, methanotrophs

## Abstract

The methane-oxidizing bacterium Methylacidimicrobium thermophilum AP8 thrives in acidic geothermal ecosystems that are characterized by high degassing of methane (CH_4_), H_2_, H_2_S, and by relatively high lanthanide concentrations. Lanthanides (atomic numbers 57 to 71) are essential in a variety of high-tech devices, including mobile phones. Remarkably, the same elements are actively taken up by methanotrophs/methylotrophs in a range of environments, since their XoxF-type methanol dehydrogenases require lanthanides as a metal cofactor. Lanthanide-dependent enzymes seem to prefer the lighter lanthanides (lanthanum, cerium, praseodymium, and neodymium), as slower methanotrophic/methylotrophic growth is observed in medium supplemented with only heavier lanthanides. Here, we purified XoxF1 from the thermoacidophilic methanotroph Methylacidimicrobium thermophilum AP8, which was grown in medium supplemented with neodymium as the sole lanthanide. The neodymium occupancy of the enzyme is 94.5% ± 2.0%, and through X-ray crystallography, we reveal that the structure of the active site shows interesting differences from the active sites of other methanol dehydrogenases, such as an additional aspartate residue in close proximity to the lanthanide. Nd-XoxF1 oxidizes methanol at a maximum rate of metabolism (*V*_max_) of 0.15 ± 0.01 μmol · min^−1^ · mg protein^−1^ and an affinity constant (*K_m_*) of 1.4 ± 0.6 μM. The structural analysis of this neodymium-containing XoxF1-type methanol dehydrogenase will expand our knowledge in the exciting new field of lanthanide biochemistry.

## INTRODUCTION

The ability to oxidize methane (CH_4_) is found in a wide range of bacterial and archaeal taxa (1, 2). Methanotrophs make a living from the oxidation of methane by using O_2_ as the terminal electron acceptor or through anaerobic respiration using a range of alternative electron acceptors (1, 3, 4). For a long time, methanotrophic diversity was thought to be restricted to the phylum Proteobacteria (5). In recent years, enrichments and isolates acquired from various environments have expanded the methanotrophic world by enabling discovery of denitrifying methane oxidizers of the NC10 phylum and nitrate- and metal-oxide-driven methane oxidation by archaea (6–9). In fact, in some environments, anaerobic methane oxidation might actually constitute the dominant methane sink (10). In addition, very acidophilic methanotrophs of the phylum Verrucomicrobia are known to thrive in acidic geothermal environments (11, 12). These verrucomicrobial methanotrophs are extremophiles, as several strains can even grow below a pH of 1 and at temperatures up to 60°C (11–13). In addition, they could be involved in various biogeochemical cycles, as they metabolize a variety of environmentally relevant molecules, such as higher alkanes, H_2_, and N_2_ (2, 14–17). Verrucomicrobial methanotrophs of the genus Methylacidiphilum have optimum growth temperatures of 50 to 60°C, whereas Methylacidimicrobium strains grow optimally at 30 to 50°C. Recently, molecular evidence demonstrated the presence of a third genus, Methylacidithermus (18). Understanding how microorganisms are involved in the carbon cycle and in methane dynamics in the environment is important, since methane is a potent greenhouse gas and its concentration in the atmosphere is increasing (19).

Aerobic methanotrophs oxidize methane to methanol (CH_3_OH) using the membrane-bound particulate methane monooxygenase (pMMO) or the cytoplasmic soluble methane monooxygenase (sMMO) (20). The product of this conversion is subsequently oxidized by a methanol dehydrogenase (MDH). This enzyme uses pyrroloquinoline quinone (PQQ) as a prosthetic group, to which a calcium ion is bound at the active site. The canonical calcium-dependent MxaFI-type MDH catalyzing this reaction consists of a large (MxaF) and a small (MxaI) subunit forming a heterotetrameric complex (21, 22). In several methylotrophs, such as the soil-inhabiting taxon Methylorubrum extorquens (formerly known as Methylobacterium extorquens), gene homologs of *mxaF* were detected through genome sequencing that were subsequently annotated as *xoxF* (23, 24). The exact role of the *xoxF* genes remained elusive for several years, but the inability of mutant strains with *xoxF* gene deletions to grow on methanol warranted further investigation (25, 26). Eventually, *xoxF* was shown to encode a quinoprotein methanol dehydrogenase that is not dependent on calcium, but rather on a lanthanide (27, 28). Lanthanides comprise a group of 15 elements with atomic numbers 57 to 71 that were for a long time considered biologically inert (29, 30). The environmental relevance of lanthanide-dependent methylotrophy became evident when the acidophilic methanotroph Methylacidiphilum fumariolicum SolV, isolated from a volcanic mud pot and only possessing a XoxF-type methanol dehydrogenase, was unable to grow in laboratory cultures without the addition of mud pot water to the growth medium (28). Inductively coupled plasma mass spectrometry (ICP-MS) analyses revealed high concentration of lanthanides in mud pot water, which lead to the finding that lanthanides are the determining growth factor for this strain. Purification and subsequent crystallization of XoxF from this strain indeed revealed a lanthanide ion in the active site, confirming the surprising role of lanthanides as metal cofactor in biology (28).

Both the calcium-dependent MxaFI-type and the lanthanide-dependent XoxF-type MDH contain pyrroloquinoline quinone (PQQ) as a cofactor (31). Interestingly, XoxF does not form a complex with a small subunit, but forms a homodimer (28). XoxF seems to be catalytically more efficient than the calcium-dependent MDH, which is thought to be the result of improved PQQ activation by the lanthanide ion (28, 32, 33). XoxF-type MDHs are currently categorized into five different types (XoxF1 to XoxF5) that are phylogenetically related to the MxaFI-type MDH (34) Crystallization of an XoxF2-type methanol dehydrogenase purified from the thermoacidophilic methanotroph *Methylacidiphilum fumariolicum* SolV revealed a high degree of structural conservation between the active sites of the XoxF-type and MxaFI-type MDHs (28). One striking difference is the additional aspartate residue in the active site of all XoxF-type MDHs that is absent in MxaFI-type MDHs (28). This specific amino acid was shown to be essential for the coordination of the lanthanide ion and therefore essential for enzyme activity (35). The exact route that electrons, derived from methanol oxidation, travel in the protein is unclear, but both MxaFI and XoxF ultimately donate their electron to a dedicated *c*-type cytochrome (36–39). Still, there is considerable evidence that the disulfide ring above the PQQ cofactor, present in alcohol-oxidizing quinoproteins such as XoxF, is involved in electron transfer to its physiological cytochrome redox partner (40). Subsequently, electrons could be transferred from the redox partner to a cytochrome *c* oxidase. *xoxF* genes are found in a large variety of environments and are divided into five types, while MxaF is thought to have evolved from a XoxF prototype (31, 34). All known methanol-oxidizing microorganisms that possess MxaFI also harbor XoxF, while many other methylotrophs only possess XoxF (41). In microbes that can make use of both proteins, expression is primarily regulated by the concentration of lanthanides, referred to as the lanthanide switch (30, 42–45).

We are becoming increasingly aware that not all 15 lanthanides result in similar growth rates in methylotrophs (30). *Methylacidiphilum fumariolicum* SolV growing in medium supplemented with either 250 nM lanthanum (La), cerium (Ce), neodymium (Nd), or praseodymium (Pr) has a growth rate of 0.085 h^−1^, whereas the growth rates in medium supplemented with either samarium (Sm), europium (Eu), or gadolinium (Gd) were determined to be 0.058, 0.038, and 0.025 h^−1^, respectively (28). The lighter elements (early lanthanides) result in high methanol-oxidizing activity whereas the heavier elements (later lanthanides) do not (46). Biochemical studies in recent years revealed how lanthanides are acquired by several microorganisms, but whether a common mechanism exists is unknown (47). Moreover, lanthanides have been shown to be involved in enzymes oxidizing multicarbon compounds, and even in enzymes in nonobligatory methylotrophs (48–50). All known *Methylacidimicrobium* strains contain both a XoxF1-type and a XoxF2-type MDH (2). In *Methylacidimicrobium thermophilum* AP8, only the XoxF1-type is highly expressed at the maximum growth rate (μ_max_) (51). Here, we report the purification and characterization of the XoxF1-type MDH from the acidophilic methanotroph *Methylacidimicrobium thermophilum* AP8. We also present the first structure of a functional neodymium-containing enzyme. In addition, it is the first structure of an MDH of the XoxF1-type. Interestingly, several amino acid residues in the active site of this neodymium-containing XoxF1-type MDH differ from those of other MDHs, which could give us clues on the evolution of the use of lanthanides as a metal cofactor in biology.

## RESULTS

**FIG 1:**
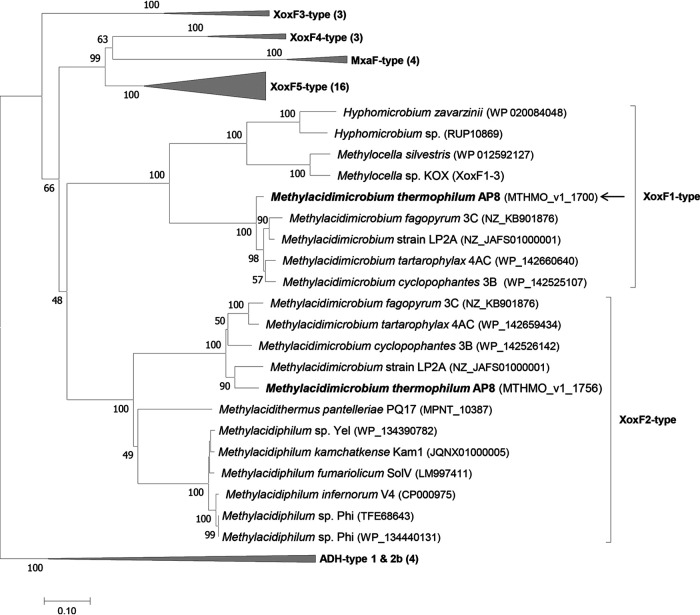
Phylogenetic tree of methanol dehydrogenases. The evolutionary history was inferred using the neighbor-joining method. The optimal tree with a sum of branch length of 8.78582079 is shown. The percentages of replicate trees in which the associated taxa clustered together in the bootstrap test (500 replicates) are shown next to the branches. The tree is drawn to scale, with branch lengths in the same units as those of the evolutionary distances used to infer the phylogenetic tree. The evolutionary distances were computed using the Jones-Taylor-Thornton (JTT) matrix-based method and are in the units of the number of amino acid substitutions per site. The analysis involved 51 amino acid sequences. All ambiguous positions were removed for each sequence pair. In total, there were 717 positions in the final data set. Alcohol dehydrogenases of the ADH type (1 and 2b) were used as an outgroup. The arrow points to the purified protein. Evolutionary analyses were conducted in MEGA 7 (79).

In the genome of the acidophilic methanotroph *Methylacidimicrobium thermophilum* AP8, two genes encoding XoxF methanol dehydrogenases of two different types were detected (51). The enzymes encoded by MTHMO_v1_1700 (*xoxF1*, type 1) and MTHMO_v1_1756 (*xoxF2*, type 2) have only 49% amino acid sequence similarity. All known *Methylacidimicrobium* strains possess both a XoxF1 and a XoxF2-type MDH, whereas *Methylacidiphilum* strains only possess a gene encoding a XoxF2-type (Fig. 1). When grown at the maximum growth rate (μ_max_) on CH_4_ and CO_2_, *xoxF1* is one of the most highly transcribed genes, whereas *xoxF2* is barely expressed (51).

By cultivating M. thermophilum AP8 in medium supplemented with 0.5 μM Nd_2_O_3_ as the sole lanthanide at μ_max_ (0.051 h^−1^) on methane, we aimed to study a functional neodymium-containing methanol dehydrogenase enzyme and determine the structure of the catalytic site. The neodymium concentration used here is in line with concentrations occurring naturally in mud pots, as determined previously (28) through inductively coupled plasma mass spectrometry (ICP-MS). The straightforward protocol developed by Pol et al. (28) used for the purification of XoxF2 from *Methylacidiphilum fumariolicum* SolV was subsequently used to purify Nd-XoxF1 from *M. thermophilum* AP8. The purification procedure resulted in a clear single dominant band on a denaturing gel (see Fig. S2 in the supplemental material). To determine whether neodymium was indeed incorporated as a metal cofactor by XoxF1, ICP-MS was performed. A neodymium occupancy of 94.5% ± 2.0% was measured.

10.1128/mBio.01708-21.3 FIG S2 SDS-polyacrylamide gel showing the purification of Nd-XoxF1 from *Methylacidimicrobium thermophilum* AP8. Lane 1, 3 μl PageRuler Plus prestained protein ladder of different masses (in kDa). Lane 2, 2 μg purified Nd-XoxF1 stained with Coomassie brilliant blue. Download FIG S2, PDF file, 0.07 MB. Copyright © 2021 Schmitz et al. 2021 Schmitz et al. https://creativecommons.org/licenses/by/4.0/ This content is distributed under the terms of the Creative Commons Attribution 4.0 International license.

**FIG 2:**
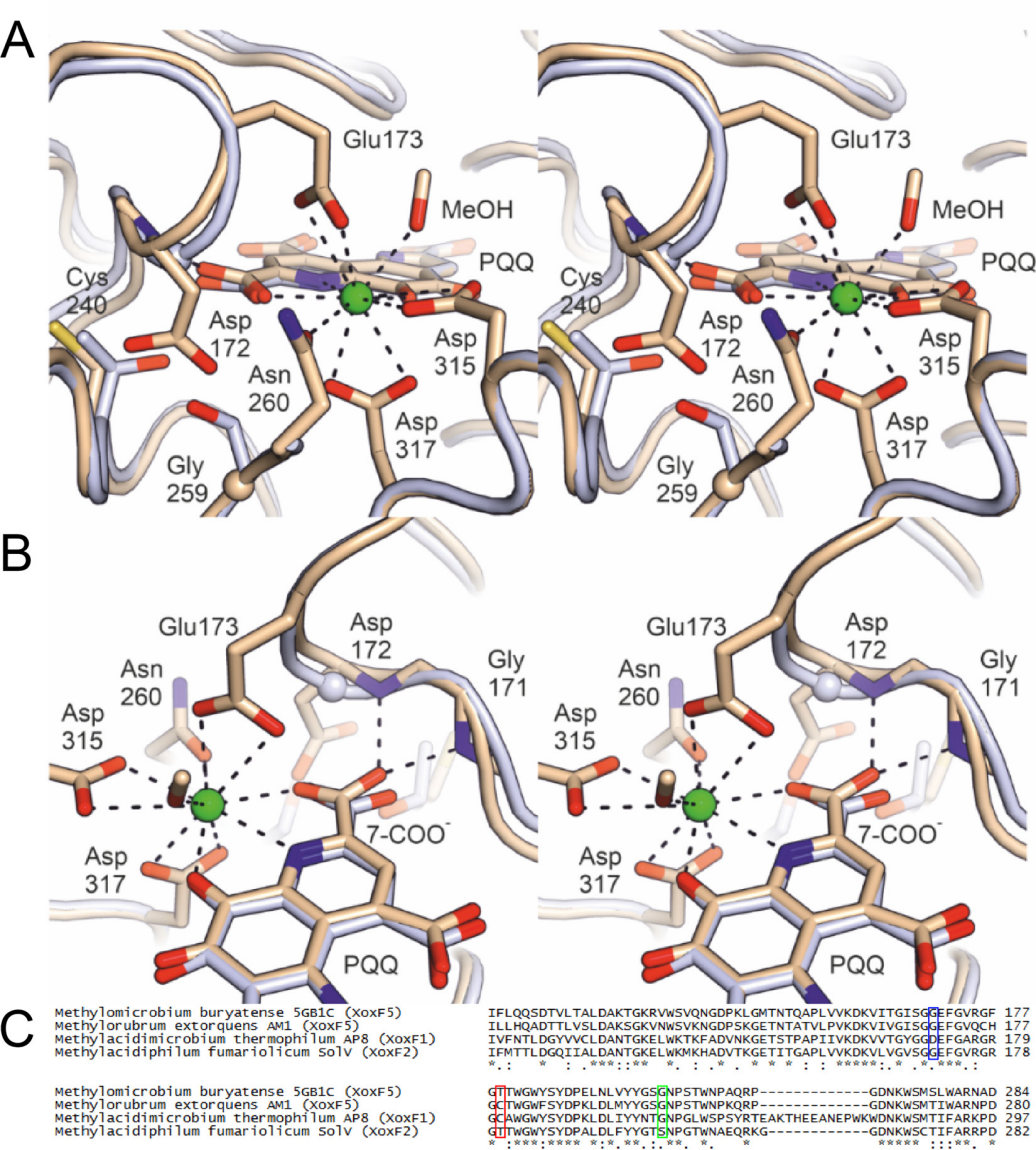
(A) Stereofigures showing the active site of the B monomer in the crystal structure of the Nd-XoxF1 dimer (beige). Numbering of amino acid residues is according to the sequence of XoxF1 of *M. thermophilum* AP8. The structure of the cerium-containing XoxF2 of *Methylacidiphilum fumariolicum* SolV (light blue) is overlaid. Relevant residues and the PQQ molecule are shown as sticks, with glycine residues shown as spheres. The Nd atom is shown as a green sphere. The diagnostic Asp172 in Nd-XoxF1 is a glycine in the XoxF2 structure, as in all other MDHs of known structure. Opposite the Asp172 side chain, Nd-XoxF1 has a cysteine at position 240 and a glycine at position 259, which in the cerium-containing XoxF2 are a threonine and a serine, respectively. (B) As shown in panel A, but in a different orientation, providing a view of the loop containing the lanthanide-coordinating residue Glu173 (present in all known XoxF structures). The presence of the bulky Asp172 in this loop causes it to adopt a different conformation compared to XoxF2 of *Methylacidiphilum fumariolicum* SolV, affecting both Glu173 and the backbone amide N-H groups of Gly171 and Asp172, which engage in hydrogen bonds with the 7-carboxylate group of PQQ in Nd-XoxF1. This carboxylate, in turn, also coordinates the lanthanide. The alignment below compares the amino acid sequences near the active site of four XoxF-type MDHs of which the crystal structure was determined. Blue frame, Asp172 of *M. thermophilum* AP8 characteristic for XoxF1-type MDHs in contrast to glycine in the other structure; red frame, Cys240 of *M. thermophilum* AP8 to make space for the bulky Asp172 residue; green frame, Gly259 close to the active site which is a serine residue in the XoxF2 structure of *Methylacidiphilum fumariolicum* SolV. Asterisks indicate conserved amino acid residues; colons and dots indicate amino acid residues with highly and weakly similar properties, respectively.

The crystal structure of Nd-XoxF1 contains a dimer in the asymmetric unit, of which one monomer contains no PQQ molecule and the other contains a PQQ molecule and neodymium ion with approximately 70% occupancy. The loss of the PQQ cofactor from the enzyme is interesting and could be a combined result of the exchange of buffers and the high salt concentration used during the crystallization procedure. In XoxF of Methylorubrum extorquens AM1, loss of PQQ was observed as well (35). The two monomers show the typical eight-bladed β-propeller fold of a methanol dehydrogenase, a conserved disulfide bridge above the PQQ cofactor and an aspartate residue coordinating the lanthanide in the active site (35, 40). They can be superimposed with the structure of the cerium-containing XoxF2 of *Methylacidiphilum fumariolicum* SolV (28) to a root mean square deviation (RMSD) of 0.8 Å. Moreover, the position and orientation of the two monomers in the dimer are identical to those in other MDH structures. However, a detailed comparison of the Nd-XoxF1 structure with those of other XoxF-type and MxaFI-type MDHs reveals an interesting difference in the direct vicinity of the active site, namely, position 172, which directly precedes the Nd-coordinating Glu173, is occupied by an aspartate residue in Nd-XoxF1 (Fig. 2), whereas this residue is a glycine in all other known MDH structures. Amino acid sequence alignments of a large variety of methanol and alcohol dehydrogenases reveals that the presence of an aspartate residue in this position is diagnostic for the XoxF1 lineage (Fig. S3). To accommodate the Asp172 side chain, residue 240 is a cysteine in Nd-XoxF1, whereas it is a bulkier threonine in XoxF2 of *M. fumariolicum* SolV. With respect to the XoxF2 structures of *Methylacidiphilum fumariolicum* SolV (28, 33), the presence of Asp172 (replacing glycine) results in a rearrangement of the loop containing the metal-coordinating Glu173 (Fig. 2A). As such, the backbone N-H groups of the loop interact with the carboxylate group of PQQ attached to C7 via H bonds (Fig. 2B). Asp315 and Asp317 in the presented structure coordinating the lanthanide correspond to Asp299 and Asp301, respectively, in the XoxF2 structure of *M. fumariolicum* SolV, which are overlaid and are therefore not visible in the stereofigures (Fig. 2)

10.1128/mBio.01708-21.4 FIG S3 Multiple-sequence alignment of various methanol and alcohol dehydrogenases. Download FIG S3, PDF file, 0.04 MB. Copyright © 2021 Schmitz et al. 2021 Schmitz et al. https://creativecommons.org/licenses/by/4.0/ This content is distributed under the terms of the Creative Commons Attribution 4.0 International license.

**FIG 3:**
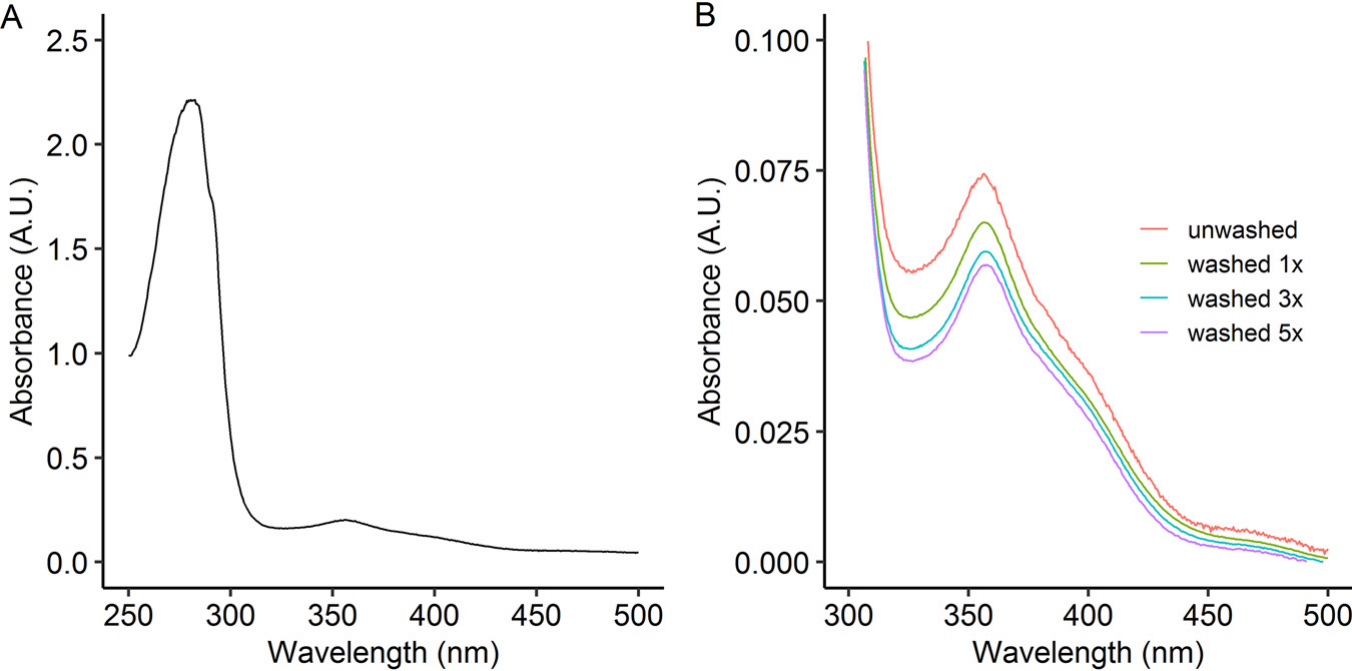
(A) UV-visible (UV-Vis) spectra of Nd-XoxF1 purified from *Methylacidiphilum thermophilum* AP8. (B) Characteristic features of the redox cofactor PQQ showing a peak at 356 nm and a shoulder at 400 nm. Washing multiple times in 20 mM PIPES buffer (pH 7.2) resulted in an increase in *A*_280_/*A*_356_ ratio due to dissociation of the PQQ cofactor.

The UV-visible (UV-Vis) spectrum of the isolated Nd-XoxF1 is very similar to other isolated XoxF proteins (28, 33) (Fig. 3A). The spectrum with absorbance maxima at 356 and 400 nm is typical for a PQQ cofactor. Since we noticed that PQQ occupancy decreased during the crystallization procedure, we investigated the stability of PQQ coordination in the active site by washing the enzyme through spin filters. With ongoing washing steps, the PQQ signal clearly decreased (Fig. 3B). The ratio of *A*_280_/*A*_356_ measured increased from 13.5 for the unwashed sample to 17.6 for the enzyme that was washed five times. The stable absorbance at 280 nm and the decrease in absorbance at 356 nm through washing indicates stability of the enzyme but dissociation of the PQQ cofactor, respectively. Similar behavior was observed with the europium-containing XoxF2 isolated from *Methylacidiphilum fumariolicum* SolV (33). The theoretical extinction coefficient of Nd-XoxF1 was determined to be 164.58 mM^−1^ cm^−1^ (33). After determination of the protein concentration by the bicinchoninic acid assay with bovine serum albumin (BSA) as the protein standard, an extinction coefficient λ_280_ of 172 mM^−1^ cm^−1^ was measured, close to the theoretical value.

**FIG 4:**
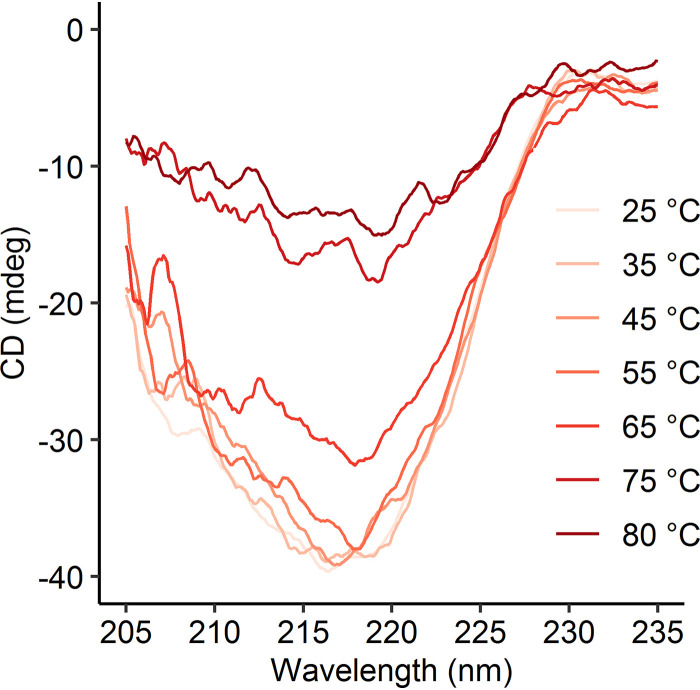
Circular dichroism spectra from 205 to 235 nm of purified Nd-XoxF1 in 20 mM potassium phosphate buffer (pH 7.0), measured at 25 to 80°C.

*M. thermophilum* AP8 is the strain within the genus *Methylacidimicrobium* with the highest optimum growth temperature (50°C), and it has a maximum growth temperature of 55°C (51). To observe the temperature-dependent stability of Nd-XoxF1, circular dichroism spectra were measured at different temperatures (Fig. 4). The spectra recorded at 25 to 55°C are highly similar, but above these temperatures, the signal distorts, indicating denaturation. Since XoxF2 of *Methylacidiphilum fumariolicum* SolV was most stable at 45°C, this temperature was chosen for kinetic comparisons.

**FIG 5:**
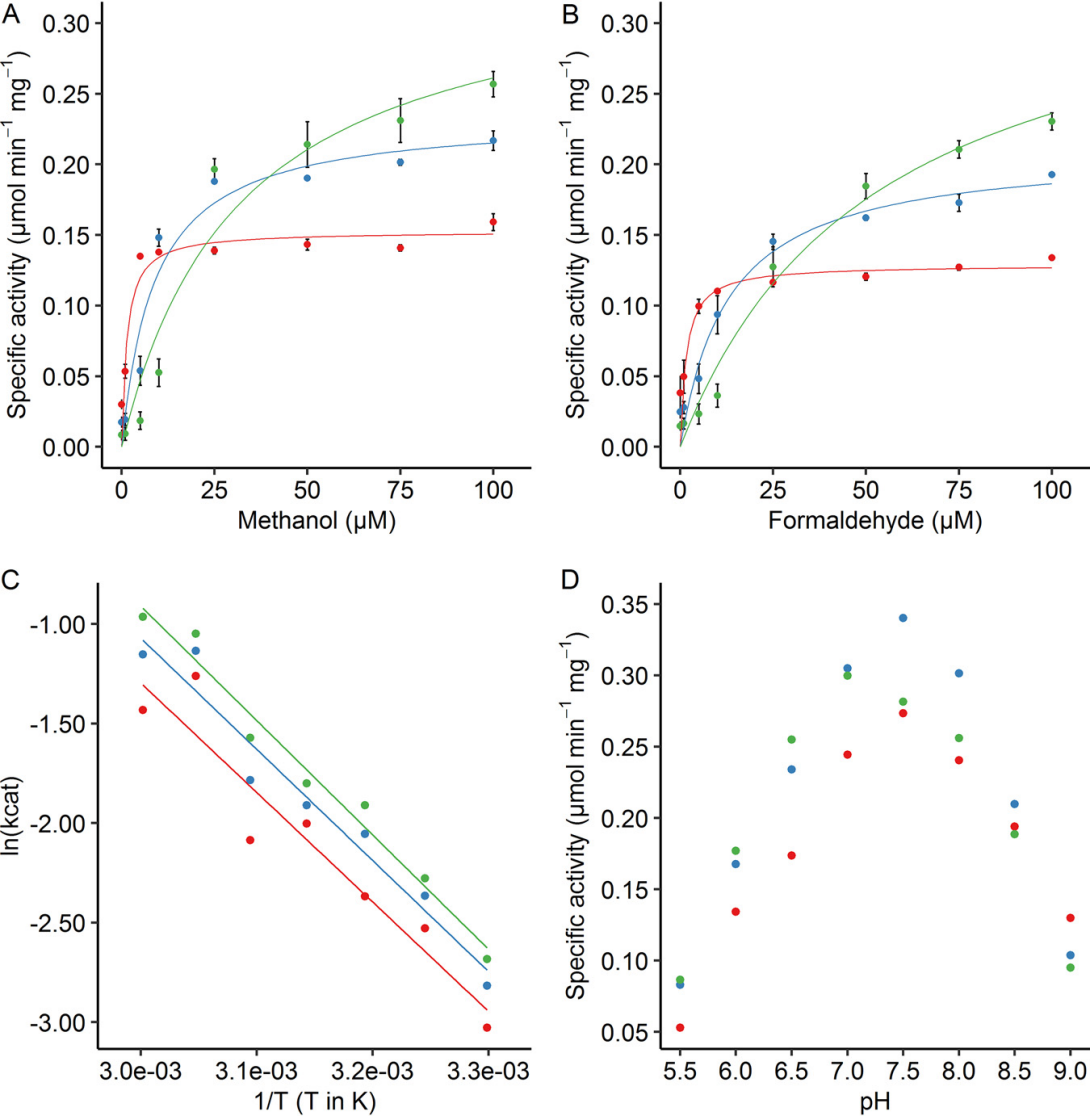
Enzyme activity of Nd-XoxF1 measured spectrophotometrically in the first 2 min after substrate addition (red dots), after 4 to 6 min (blue dots) and after 8 to 10 min (green dots). Specific enzyme activities at different concentrations of methanol (A) or formaldehyde (B). Data points represent averages of duplicates, error bars represent standard deviations. (C) Arrhenius activation energy for methanol oxidation at different temperatures. Data points represent averages of duplicates. (D) Specific enzyme activities on methanol at different pH values.

Initial activity assays at pH 7 and pH 9 with and without NH_4_^+^ revealed low activity at pH 9 and high activity at pH 7, with a slight increase in activity upon NH_4_^+^ addition. Classically, ammonium is added to artificial enzyme assays with calcium-dependent MxaFI-type MDH for methanol oxidation to proceed (52). Since in our case methanol oxidation proceeds without addition of ammonium, albeit at a slightly lower rate, ammonium was omitted from the assay. Addition of NdCl_3_ did not significantly enhance activity any further, which is logical considering the high (94.5% ± 2.0%) occupancy of Nd measured through ICP-MS. Activity assays with 2,6-dichlorophenolindophenol (DCPIP)/phenazine ethosulfate (PES) performed in an Epoch 2 plate reader using 96-well plates revealed that Nd-XoxF1 catalyzes methanol oxidation with a maximum rate of metabolism (*V*_max_) of 0.15 ± 0.01 μmol · min^−1^ · mg protein^−1^ and an affinity constant (*K_m_*) of 1.4 ± 0.6 μM. Formaldehyde oxidation kinetics were determined at 0.13 ± 0.01 μmol · min^−1^ · mg protein^−1^ and a *K_m_* of 1.6 ± 0.8 μM. Interestingly, Nd-XoxF1 presents a lag phase in kinetics, which is probably artificial. After 3 min, the activity increases, while the affinity decreases (Fig. 5A and B). Hence, the time frame at which the slope is determined is important. When the activity is measured in the first 2 min, the data points give the best fit in Michaelis-Menten kinetics. To assess the lag phase in kinetics that was observed, the assay was repeated in a Cary spectrophotometer using a 4-ml stirred glass cuvette. This time, no lag phase was detected and a *K_m_* of 1.5 ± 0.1 μM was calculated, in agreement with what was observed above (Fig. S4). The observed difference in lag phase could be due to the methodology. For example, the solutions in a 96-well plate and glass cuvettes are heated and stirred in different ways. In addition, the 96-well plates are made of plastic instead of glass, which might cause differences. All in all, it is important to measure rates directly after addition of methanol for the most realistic values.

10.1128/mBio.01708-21.5 FIG S4 Specific enzyme activity of methanol oxidation by Nd-XoxF1 measured spectrophotometrically in a 4-ml cuvette. Download FIG S4, PDF file, 0.07 MB. Copyright © 2021 Schmitz et al. 2021 Schmitz et al. https://creativecommons.org/licenses/by/4.0/ This content is distributed under the terms of the Creative Commons Attribution 4.0 International license.

To assess the temperature dependence, the Arrhenius activation energy was determined at a value between 45.1 to 47.8 kJ · mol^−1^ (Fig. 5C), while the pH optimum was determined at 7.5 (Fig. 5D). These values are similar to previously reported values of the europium-containing XoxF2 from *Methylacidiphilum fumariolicum* SolV (53).

## DISCUSSION

In this study, we characterized a novel XoxF1 methanol dehydrogenase that incorporates the element neodymium as metal cofactor for methanol oxidation. The discovery of the lanthanide dependency of XoxF and the structural analysis of cerium-containing XoxF2 initiated a new field in which lanthanides are investigated as metal cofactors in biology (27, 28, 54). By growing the thermoacidophilic methanotroph *Methylacidimicrobium thermophilum* AP8 with neodymium added to the growth medium, we managed to purify a neodymium-containing XoxF1-type MDH. The crystal structure determined is the first structure of a XoxF1-type MDH and revealed interesting differences in amino acid composition of the active site compared to those of other methanol dehydrogenases. Most importantly, an additional aspartate residue (Asp172 in the structure presented here) seems to be diagnostic for XoxF1-type MDHs. The backbone N-H group of this residue interacts with the carboxylate group of PQQ attached to C7 via H bonds, which do not seem to lead to differences in kinetics properties. To enable the large space that Asp172 occupies, the bulky threonine residue found in XoxF2 of *M. fumariolicum* SolV is replaced by a relatively small cysteine residue. The coordination of PQQ and the lanthanide are almost identical to the coordination in other XoxF-type MDHs, and therefore the overall coordination of the lanthanide is not altered in XoxF1 compared to that of other XoxF-type MDHs. Hence, multiple ways seem to have evolved to obtain very similar cofactor coordination in XoxF methanol dehydrogenases.

Despite recent genomic analyses revealing an enormous diversity of potential lanthanide-dependent enzymes in various environments, the use of lanthanides in biology is still largely unexplored (50). The concentration of neodymium that was added to the growth medium is typical for acidic geothermal ecosystems (28, 55). Lanthanides are quite available in these ecosystems, due to the increased solubility of lanthanides at low pH. Other environments in which *xoxF* genes were detected, such as soil, oceans, and lakes, are characterized by significantly lower concentration of lanthanides, typically in the picomolar or nanomolar range (55). Iron availability from these environments is low as well, which is overcome by using siderophores (56). Similarly, the methylotroph Methylorubrum extorquens seems to secrete a chelator for the uptake of lanthanides (57, 58). This bacterium possesses a gene encoding a 12-kDa periplasmic protein with highly selective lanthanide-binding capacity that was named lanmodulin (59, 60). The gene adjacent to the gene encoding lanmodulin encodes a TonB-dependent transporter that was shown to be involved in lanthanide uptake (58, 61). Interestingly, none of these genes involved in lanthanide uptake can be found when sequences are subjected to a BLAST search against the genome sequences of *Methylacidimicrobium thermophilum* AP8 and other verrucomicrobial methanotrophs. Possible explanations could be that utilization of lanthanides started in acidic geothermal ecosystems in which such complex uptake mechanisms were not needed. To colonize other niches, the need for a more advanced machinery for lanthanide acquisition may have evolved.

The 85-fold higher expression of *xoxF1* versus *xoxF2* under maximum-growth conditions is remarkable (51). In microorganisms that harbor both *mxaF* and *xoxF*, it is known that lanthanides respectively repress expression of *mxaF* and induce expression of *xoxF* (58, 62). Interestingly, when the methanotroph Methylobacter tundripaludum strain 31/32 was grown in the presence of a nonmethanotroph methylotroph, gene expression shifted from *xoxF* to *mxaF* (63). MxaF has a lower affinity for methanol than XoxF and, as such, more methanol produced by the methanotroph could be excreted and subsequently utilized by the methylotrophic partner (31, 63). Which environmental factors regulate the expression of different copies of *xoxF* is currently unknown. *Methylacidiphilum* strains that possess multiple operons encoding pMMO are known to differentially express these genes under various oxygen-limiting or nitrogen-fixing conditions (64). Since XoxF was shown to utilize different carbon compounds, substrate availability might regulate gene expression of multiple *xoxF* genes (28).

The neodymium occupancy of Nd-XoxF1 studied here was determined through ICP-MS to be almost 100%. For the XoxF2 of *Methylacidiphilum fumariolicum* SolV grown in medium supplemented with europium, the europium occupancy of the active site was only 70% (33). The 15 lanthanides are not used evenly by enzymes as a metal cofactor, as a clear preference is observed for the early lanthanides lanthanum (La), cerium (Ce), praseodymium (Pr), and neodymium (Nd) (65). The methanotroph *Methylacidiphilum fumariolicum* SolV was shown to have similar growth rates on either one of these early lanthanides, but the growth rate decreased gradually when medium was supplemented with samarium (Sm), europium (Eu), and gadolinium (Gd) instead (28). The growth rate on Gd was about 30% compared to the early lanthanides. Additions of La^3+^ or Pr^3+^ to the partially occupied apo-europium-containing XoxF2 of *Methylacidiphilum fumariolicum* SolV resulted in increased catalytic efficacies (33). This could explain why *M. fumariolicum* SolV has a higher growth rate on lanthanum and praseodymium compared to that on europium. In addition, a higher binding affinity between XoxF and XoxG in *Methylorubrum extorquens* AM1 is observed when XoxF has the lightest lanthanide lanthanum incorporated in comparison to those with heavier lanthanides such as neodymium (38, 66). Washing cycles of Nd-XoxF1 revealed PQQ dissociation, which could also lead to wash-out of the lanthanide. Moreover, the crystals of Nd-XoxF1 showed a loss of the PQQ cofactor, which could be due to the high concentration of salt used in the procedure. Whether loss of PQQ and lanthanides is a result of *in vitro* conditions or a characteristic of certain types of lanthanide-dependent methanol dehydrogenases remains to be determined (35).

The kinetic properties determined for Nd-XoxF1 show clear similarities and differences to those of other XoxF methanol dehydrogenases. The affinity constants (*K_m_*) for methanol of different XoxF-type MDHs varies remarkably from 0.8 to 55 μM (67). What makes comparison complicated is the fact that the numbers assigned to XoxF-type MDHs not always refer to the XoxF type, as the XoxF of clade/type 5 was named XoxF1 because it is one of two XoxF MDHs of *Methylorubrum extorquens* AM1 (68). The XoxF MDHs can be divided into those with a relatively high affinity (0.8 to 3.6 μM methanol) of types XoxF1 and XoxF2 (including the XoxF1 studied here) (28, 33, 46), and those with a relatively low affinity (29 to 55 μM methanol) of types XoxF4 and XoxF5 (68–70). Still, the conditions for the activity assays used for different XoxF-type MDHs vary greatly in addition to experimental difficulties, such as background reactions and instability of the artificial electron acceptors (71). Hence, values can often not be directly compared. XoxF4 is found in Methylophilaceae species, while XoxF5 is found in a variety of proteobacterial taxa (70). Another important difference is that the pH optimum of XoxF4 and XoxF5 is around 9, similar to that of MxaF MDHs, although this is mostly artificial and assay dependent (72). These dissimilarities might also be the result of different properties of various amino acids near the active site. All known XoxF methanol dehydrogenases oxidize various alcohols with similar rates compared to that for methanol (28, 70). The differences in catalytic properties and active site structures might help to resolve the role and evolution of different XoxF types in various environments.

In conclusion, we expanded knowledge in the exciting and rapidly developing field of lanthanide biochemistry by the purification of a Nd-XoxF1-type MDH from a thermoacidophilic methanotroph. For the first time, we show that neodymium is incorporated into the active site of XoxF1. Although the amino acid composition of the active site shows clear structural differences compared to those of other XoxF-type MDHs, the coordination of the PQQ cofactor and lanthanide is very similar.

## MATERIALS AND METHODS

### Growth of *Methylacidimicrobium thermophilum* AP8 and medium composition.

*Methylacidimicrobium thermophilum* AP8 was isolated from geothermal soil on the island of Pantelleria and grown in a chemostat batch culture on methane at maximum growth rate (μ_max_) (see Fig. S1 in the supplemental material) with Pantelleria medium, as described previously (51). Neodymium was the only lanthanide that was supplemented, to a final concentration of 0.5 μM Nd_2_O_3_. The biomass used for purification of XoxF was harvested after a batch phase of about 7 days. NH_4_Cl and trace element solutions were manually added to the culture to reach an optical density at 600 (OD_600_) of 10.

10.1128/mBio.01708-21.2 FIG S1 Growth curve of Methylacidimicrobium thermophilum AP8 in a batch chemostat. Download FIG S1, PDF file, 0.01 MB. Copyright © 2021 Schmitz et al. 2021 Schmitz et al. https://creativecommons.org/licenses/by/4.0/ This content is distributed under the terms of the Creative Commons Attribution 4.0 International license.

### Purification of Nd-XoxF1 from *M. thermophilum* AP8.

To purify native Nd-XoxF1 from *M. thermophilum* AP8 biomass, cells from the above-described culture were harvested by centrifugation (15 min at 5,000 × *g* and 4°C). The supernatant was discarded and the cell pellet was resuspended in 10 mM piperazine-*N*,*N*′-bis(2-ethanesulfonic acid (PIPES) buffer (pH 7.2) supplemented with 30 mg · liter^−1^ DNase I. To lyse the cells, the concentrated cell suspension was passed three times through a French pressure cell press at 120 MPa (American Instrument Company, Silver Spring, MD). To separate the soluble proteins from the membrane proteins, the crude extract was centrifuged in an ultracentrifuge (1 h at 140,000 × *g* and 4°C). The supernatant was taken and centrifuged again at the same speed to pellet any remaining membranes. Subsequently, the supernatant was used for protein purification. Nd-XoxF1 was purified using an isolation procedure described previously (28). The buffer used during the procedure contained 1 mM methanol, which is necessary to minimize loss of enzymatic activity.

### SDS-PAGE.

The purity of Nd-XoxF1 was assessed on home-made 10% SDS polyacrylamide gels. As a molecular weight reference, 3 μl PageRuler Plus prestained protein ladder (Thermo Fisher Scientific, Walthman, MA) was used. A 2-μg aliquot of purified Nd-XoxF1 was incubated in SDS sample buffer (39) for 10 min at 100°C before being loaded on the gel. After running, the gels were stained in Coomassie brilliant blue for 1 h and destained afterwards.

### Inductively coupled plasma mass spectrometry.

To determine the occupancy of neodymium (Nd^3+^) in purified XoxF1, inductively coupled plasma mass spectrometry (ICP-MS) was performed. Protein concentrations were determined as described previously (33). Fractions of 450 μl containing 16.6 μM isolated MDH were concentrated to 45 μl in Vivaspin 500 centrifugal spin filters with a 30-kDa cutoff value (Sartorius, Germany). The concentrated protein sample was mixed with 455 μl of 11% nitric acid, and samples were destructed by heating at 90°C for 1 h. Subsequently, samples were mixed with 4.5 ml of 1% nitric acid and measured for metal content. Calibrations of 0 to 200 ppb were used for Ca, Mn, Cu, Zn, Sr, Ba, La, Ce, Pr, Nd, Sm, Eu, and Gd.

### Crystal structure determination.

Initial attempts at determining the crystal structure of Nd-XoxF1 resulted in structures without PQQ bound to the active site. We attributed this to the extensive buffer exchange performed prior to crystallization, as well as to the presence of acetate in the crystallization procedure, which we found to lead to dissociation of PQQ from the protein. We therefore performed new crystallization screens using Nd-XoxF1 (*A*_280_^1 cm^ = 27.4) in 250 mM PIPES/NaOH (pH 6.7) protein supplemented with 1 mM NdCl_3_ and 1 mM PQQ sodium salt. Optimal crystallization conditions were identified by screening in 100- + 100-nl sitting drop setups pipetted from commercial crystallization screens onto XTL low-profile plates (Greiner Bio One, Frickenhausen, Germany) by a Mosquito pipetting robot (TTP Labtech, Jena, Germany). This resulted in several hits, including a condition from the JCSG Core II screen containing 0.01 M nickel chloride, 20% (wt/vol) polyethylene glycol (PEG) 2000 monomethyl ether and 0.1 M Tris-HCl (pH 8.5). From this condition, thick plate-like crystals grew in 1 day. These crystals were cryoprotected in reservoir solution supplemented with 15% (vol/vol) ethylene glycol and flash-cooled in liquid nitrogen. A 2.3-Å resolution data set was collected at the PX-II beam line of the Swiss Light Source at the Paul Scherrer Institute in Villigen (Switzerland), which was processed using XDS software (73). These data were phased by molecular replacement with PHASER (74, 75) using the structure of the cerium-containing XoxF2-type MDH from *Methylacidiphilum fumariolicum* SolV (28) (PDB entry 4MAE). The final model was obtained by iterative rebuilding in COOT (76) and refinement in PHENIX (77, 78). To ensure that the metal ion observed in the active site is not a nickel ion from the crystallization solution, we also refined the structure with nickel ions in the metal binding site, using B-factor and occupancy refinement. This resulted in strong electron difference density around the nickel ions, B-factors that were much lower than those of the surrounding atoms, and occupancies higher than those of the PQQ molecule in the A molecule. When neodymium ions were placed in these sites, only minimal difference density was observed around the Nd ions, their B-factors were close to those of the surrounding atoms, and their occupancy refined to a value very close to that obtained for the PQQ molecule. We therefore conclude that the ions in the metal binding sites are neodymium ions. Data and model statistics are reported in Table S1 and have been submitted to the PDB under accession number 7O6Z.

10.1128/mBio.01708-21.1 TABLE S1 Diffraction data and crystallographic model statistics. Values for the highest-resolution shell are given in parentheses. Download Table S1, PDF file, 0.04 MB. Copyright © 2021 Schmitz et al. 2021 Schmitz et al. https://creativecommons.org/licenses/by/4.0/ This content is distributed under the terms of the Creative Commons Attribution 4.0 International license.

### UV-visible spectroscopy.

Spectra were collected using a Cary 60 spectrophotometer in a quartz microcuvette with 10-mm path length. To assess the effect of washing on dissociation of the PQQ cofactor, Nd-XoxF1 was washed with 20 mM PIPES buffer (pH 7.2Enzyme (10 μl) was diluted in buffer at a ratio of 1:10. The diluted protein sample was transferred to an equilibrated Vivaspin 500 centrifugal spin filter with a 30-kDa cutoff value (Sartorius) and centrifuged at 4,000 × *g* and 4°C for 15 min. This procedure was repeated six times, and spectra were recorded. Data were baseline corrected to 600 nm and then normalized to the absorbance measured at 280 nm, representing the total protein peak.

### Circular dichroism spectroscopy.

To assess the stability of Nd-XoxF1 at different temperatures, circular dichroism (CD) spectroscopy was performed using a J-810 CD spectrometer (Jasco, Oklahoma City, OK). The enzyme was diluted in 20 mM potassium phosphate buffer (pH 7) to 11.9 μM. Potassium phosphate buffer was used because it displays significantly less background absorption compared to PIPES buffer at a wavelength of 200 to 250 nm. In a 2-mm-path-length cuvette, the spectrum was monitored at different temperatures using an external water bath connected to the circular dichroism cell compartment. Scanning settings were performed as described previously (33).

### Dye-coupled enzyme kinetics.

Methanol dehydrogenase activity tests were performed in 100 mM multicomponent buffer (25 mM citric acid, 25 mM bis-Tris, 25 mM Tris, and 25 mM *N*-cyclohexyl-2-aminoethanesulfonic acid [CHES]), 1 mM phenazine ethosulfate (PES), and 100 μM 2,6-dichlorophenolindophenol (DCPIP) at pH 7 and 45°C in an Epoch 2 plate reader and 96-well plates (71). Each well contained 200 μl total volume. To minimize background reactions, an assay premixture containing 2 mM PES and 200 μM DCPIP in buffer was prepared and heated to 45°C for 15 min and then stored on ice in an amber Falcon tube to prevent light-induced degradation. 100 μl of this assay mixture was placed in each well, followed by addition of 90 μl Nd-XoxF1 in buffer to yield a final concentration of 200 nM enzyme in the assay. Methanol dilutions (10 μl) were added after the background activity had been monitored for 2 min at 45°C in the plate reader at 600 nm. The obtained data were path length corrected to 1 cm using the protocol in the Gen5 software. The activity was then monitored for 10 min, and data were fitted separately using the first 2 min, min 4 to 6, and min 8 to 10. A 1 M formaldehyde stock was prepared by dissolving paraformaldehyde powder in multicomponent buffer (pH 7.5), followed by addition of a few drops of 1 M NaOH to help with dissolution. The resulting suspension was sonicated at 50°C for 30 min until the solution was clear. The pH was then readjusted to 7.0 with 1 M HCl. Dilutions were prepared using Millipore water. In addition, to assess temperature dependence, enzyme activity was assessed at pH 7 at 30 to 60°C in 5°C steps in duplicates. To determine the enzymatic activity at different temperatures and pH values, the above-mentioned 100 mM multicomponent buffer was used. The pH dependence was assessed both with and without 15 mM NH_4_Cl.

The MDH activity assay was also performed in a 4-ml stirred glass cuvette and placed in a Cary spectrophotometer heated at 45°C. The reaction mixture was prepared as described above. The Nd-XoxF1 used in this essay was stored in 10 mM phosphate buffer supplied with 1 mM methanol. Several washing steps using 30-kDa Vivaspin centrifugal spin filters (Sartorius) were necessary to eliminate the residual methanol before the essay could be performed. The enzyme was resuspended in 100 mM multicomponent buffer and 1 mM cyanide.

### Phylogenetic analysis.

The XoxF amino acid sequences of *Methylacidimicrobium thermophilum* AP8 were used in BLASTP searches against the GenBank database. Representative homologous protein sequences were downloaded and combined with representatives from the different types of methanol dehydrogenases (31). Sequences were aligned by the ClustalW tool available in MEGA7 (79). MEGA7 was also used to infer the evolutionary history of the representative protein sequences using the neighbor-joining method.

### Data availability.

The protein structure described in this article has been deposited in the Protein Data Bank (PDB) under accession number 7O6Z.
