# Supplementary material for: Neodymium as Metal Cofactor for Biological Methanol Oxidation: Structure and Kinetics of an XoxF1-Type Methanol Dehydrogenase
Source: mBio. 2021 Sep 21;12(5):e01708-21. doi: 10.1128/mBio.01708-21 (PMC8546591; doi:10.1128/mBio.01708-21)
Supplement: FIG S2 [file mbio.01708-21-sf002.pdf]

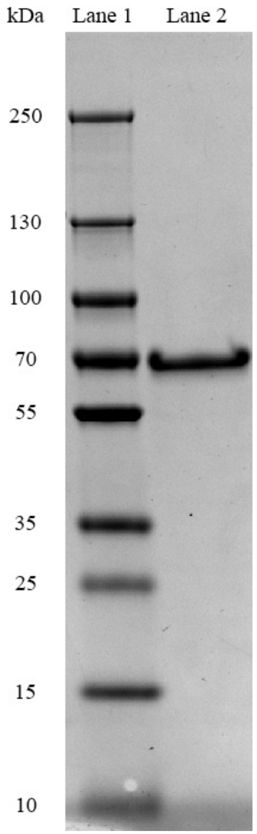

Figure S2: SDS-polyacrylamide gel showing the purification of Nd-XoxF1 from *Methylacidimicrobium thermophilum* AP8. Lane 1: 3 µL PageRuler Plus Prestained Protein Ladder protein ladder of different masses in kDa. Lane 2: 2 µg purified Nd-XoxF1 stained with Coomassie Brilliant Blue.
