# Supplementary material for: Neodymium as Metal Cofactor for Biological Methanol Oxidation: Structure and Kinetics of an XoxF1-Type Methanol Dehydrogenase
Source: mBio. 2021 Sep 21;12(5):e01708-21. doi: 10.1128/mBio.01708-21 (PMC8546591; doi:10.1128/mBio.01708-21)
Supplement: FIG S3 [file mbio.01708-21-sf003.pdf]

Figure S3: Multiple sequence alignment of various methanol and alcohol dehydrogenases.

#1_ACS42169_Methylobacterium_extorquens_AM1_MxaF-type
#2_WP_012589514.1_Methylocella_silvestris_mxaFJGIRSACKLDEH_MxaF-type
#3_xoxF_2_Methylocella_sp._KOX_MxaF-type
#39_Hyphomicrobium_VS_HYPHVSv1_1280001_MxaF-type
#4_WP_036262132.1_Methylocapsa_aurea_XoxF5-type
#5_WP_091681465.1_Methylocapsa_palsarum_XoxF5-type
#6_WP_026607385.1_Methylocapsa_acidiphila_XoxF5-type
#7_WP_012590606.1_Methylocella_silvestris_xoxFGJ_XoxF5-type
#8_ACS40517_Methylobacterium_extorquens_AM1_XoxF5-type
#9_WP_003597620.1|Methylobacterium_extorquens_XoxF5-type
#10_WP_011746370.1|Paracoccus_denitrificans_XoxF5-type
#11_xoxF_4_Methylocella_sp._KOX_XoxF5-type
#12_WP_041367950.1_Methylocella_silvestris_XoxF5-type
#34_WP_092868386.1_xoxF_Hyphomicrobium_facile_XoxF5-type
#33_WP_013215583.1_xoxF_Hyphomicrobium_denitrificans_XoxF5-type
#38_WP_092037086.1_Methylobacterium_pseudosasicola_XoxF5-type
#37_WP_091863466.1_Methylobacterium_brachiatum_XoxF5-type
#17a_AAU90462.1_Methylococcus_capsulatus_str._Bath_XoxF5-type
#13_D14_FHGOONML_01469_Uncultured_Methylococcales_XoxF5_type
#14_E33_NFPLPOKJ_01232_Uncultured_Methylococcales_XoxF5_type
#42_YP_004038451.1_Methylovorus_sp._MP688_XoxF4-type
#40_YP_003674616.1_Methylotenera_versatilis_301_XoxF4-type
#41_YP_546422.1_Methylobacillus_flagellatus_KT_XoxF4-type
#19_xoxF_1_Methylocella_sp._KOX_XoxF1-type
#17_WP_012251923.1_Methylobacterium_extorquens_XoxF3-type
#18_WP_012592359.1_Methylocella_silvestris_xoxFJG_XoxF3-type
#29_Methylacidimicrobium_fagopyrum_3C_NZ_KB901876_XoxF2-type
#31_Methylacidimicrobium_tartarophylax_4ac_XoxF2-type
#30_Methylacidimicrobium_cyclopophantes_3B_XoxF2-type
#32_NZ_JAFS01000001_Methylacidimicrobium_strain_LP2A_XoxF2-type
#20_Methylacidimicrobium_thermophilum_AP8_MTHMO_v1_1756)_XoxF2-type
#WP_134390782.1_Methylacidiphilum_sp._Yel_XoxF2-type
#TFE68643.1_Methylacidiphilum_sp._Phi_XoxF2-type
#WP_134440131.1_Methylacidiphilum_sp._Phi_XoxF2-type
#MPANT_v1_10387_Methylacidithermus_pantelleria_PQ17_XoxF-type
#25_LM997411_xoxF_Methylacidiphilum_fumariolicum_SolV_XoxF2-type
#26_CP000975_xoxF_Methylacidiphilum_infernorum_V4_XoxF2-type
#28_JQNX01000005_xoxF_Methylacidiphilum_kamchatkense_Kam1_XoxF2-type
#15_xoxF_3_Methylocella_sp._KOX_XoxF3-type
#16_WP_012592127.1_Methylocella_silvestris_xoxFJG_XoxF1-type
#35_RUP10869.1_Hyphomicrobium_sp._XoxF1-type
#36_WP_020084048.1_Hyphomicrobium_zavarzinii_XoxF1-type
#27_Methylacidimicrobium_thermophilum_AP8_(MTHMO_v1_1700)_XoxF1-type
#21_Methylacidimicrobium_cyclopophantes_3B_Xanthomonas_type_XoxF1-type
#22_NZ_JAFS01000001.1:_Methylacidimicrobium_Strain_LP2A_XoxF1-type
#23_Methylacidimicrobium_fagopyrum_3C_NZ_KB901876_XoxF1-type
#24_Methylacidimicrobium_tartarophylax_4ac_XoxF1-type
#45_YP_001203590.1_Bradyrhizobium_sp._ORS_278_ADH-type_1
#46_YP_007512722.1_Bradyrhizobium_oligotrophicum_S58_ADH-type_1
#43_YP_003067544.1_Methylobacterium_extorquens_DM4_ADH-type_2b
#44_YP_007915167.1_Hyphomicrobium_denitrificans_1NES1_ADH-type_2b

The order on the first pages is identical to the identifier in the alignment however with full accession number and identity.

At the right site of the alignment the different types of PQQ-methanol dehydrogenases is indicated.

This alignment combines the five clades of XoxF enzymes, the MxaF-type methanol dehydrogenases and alcohol dehydrogenases. In red the aspartate (D) residues that seems to be typical for the XoxF1-type. In yellow the cysteine (C) that is present to make space for the aspartate residue in XoxF1, although this cysteine residue also found in some XoxF5 MDHs.

In green the specific aspartate (D) identified to coordinate the lanthanide in the active site. The calcium-binding MxaF-type MDHs have an alanine (A) at this position.

In grey the typical CC (PQQ binding) which is as CY in the *Methylacidimicrobium* XoxF2-type sequences.

```
1_ACS42169 ---------- -N-DKLVELS KSDDNWVMPG KNYDSNNFSD LKQINKGNVK QLRPAWTFST G--LLNGHEG APLVV----- DGKMYIHTSF -P-----NNT FALGLDD--P |---
2_WP_01258 ---------- -N-DKLNQLS QSDDNWVMPG KDYKSDNFSK LTQINASNVK QLKPAWSFST G--VLNGHEG APLVV----- DGKMFISSPF -P-----NTT FALDLDE--P |
3_xoxF_2_M ---------- -N-DKLDQLS QSDENWVMPG KNYHSDNFSK LTQINADNVK DLKAAWSFST G--VLNGHEG APLVI----- DGTMYVHTSF -P-----NIT YALDLDN--P |MxaF
39_Hyphomi ---------- -N-DKLIELS KSNENWVMPG KNYDSNNYSE STQVNAENVK QLKHAWSFST G--ELHGHEG APLVI----- GDMMYVHSSF -P-----NKT FALNLND--P |___
4_WP_03626 ---------- -N-DGLIALS KDPKQWALPT GDYANRRYSE LSQITSENVK TLVPVWSFST G--VLRGHEG APLVI----- GNVMYLSTPF -P-----NIV YALDLNN--D |
5_WP_09168 ---------- -N-DELAAQS KDPKQWVLPT GDYANRRFSE LSQINSSNVK NLVPAWSFST G--VLRGHEG GPLVV----- GNVMYFSTPF -P-----NIV YALDLNN--D |
6_WP_02660 ---------- -N-DELIALS KDAKQWALPT GDYANRRYSE LDQINSDNVR NLVPVWSFST G--VLRGHEG GPLVI----- GDVMYFNTPF -P-----NII YALDLNH--D |
7_WP_01259 ---------- -N-EELITLQ KNAKNWALPT GDYSNHRFSE LNQITNKNVK DLVPVWTFST G--VLRGHEG NPLVI----- GDVMYFSTPF -P-----NIV YALDLNN--D |
8_ACS40517 ---------- -N-EDVLKRT QDPNQQVLQT LDYANTRYSK LDQINASNVK NLQVAWTFST G--VLRGHEG SPLVV----- GDIMYVHTPF -P-----NIV YALDLNN--D |
9_WP_00359 ---------- -N-ESVLKGV ANPAEQVLQT VDYANTRYSK LDQINASNVK NLQVAWTFST G--VLRGHEG SPLVV----- GNIMYVHTPF -P-----NIV YALDLDQ--G |
10_WP_0117 ---------- -N-DSVLAEI AKPQQWAIQM GDYANTRYST LDQINKDNVK DLRVAWTFST G--VLRGHEG SPLVI----- GDVMYVHTPF -P-----NRV FALDLND--N |
11_xoxF_4_ ---------- ---------- ------EEAA PDLANLRYSP LKEITTENVS RLTPVWTFST G--VLRGHEG APLVV----- GGVIYVHTPF -P-----NIV YALDLDH--D |XoxF5
12_WP_0413 ---------- ---------- ---GSAANAD SDYANLRYSS LSEINVDTVQ NLAPVWTFST G--VLRGHEG APLVA----- GGALYLVTPF -P-----NRV YALDIAH--E |
34_WP_0928 ---------- -N-RDVIAHS NDPADWAVQS GDYSGQRYSK LAEINKDNVG DLKVAWTFST G--VLRGHEG GPLVI----- GDTMYLVTPF -P-----NAV MALDLAH--D |
33_WP_0132 ---------- -N-ESAIKAA SDPNNWAIQS GDYAGTRYST LDQITTENVG QLKVAWSFST G--VLRGHEG GPLVI----- GDTMWLHSAF -P-----NIV FALNLAD--E |
38_WP_0920 ---------- ------VASA QEPP--VLQT LDYANTRYSE LAQIDAGNVS RLKVAWTFST G--VLRGHEG APIVV----- GSRMFVHTPF -P-----NLV YALDLDH--D |
37_WP_0918 ---------- ------VASA KEPP--VLQT LDYANTRYSD LAQIDAGNVS RLKVAWTFST G--VLRGHEG APIVV----- GNRMFVHTPF -P-----NLV YALDLDH--D |
17a_AAU904 ---------- -N-AEVEALT KDPKNFATWG GNYAGTRYST LDQINFKNAK HLQPVWTFST G--MLRGHEG GPLVV----- NDVIYIHTGY -P-----HKV YALDQA---T |
13_D14_FHG ---------- -N-SGVESLT KNPANWATAL GDYAGTRYSK LDQINTKNVK NLQPAWSFST G--VLRGHEG GPLVV----- DDIIYIHTPF -P-----NTV YALDQA---T |
14_E33_NFP ---------- -N-SGVESLT KNPANWATAL GDYAGTRYSK LDQINTKNVK NLQPAWSFST G--VLRGHEG GPLVV----- DDIIYIHTPF -P-----NTV YALDQA---T |___
42_YP_0040 ---------- -A-TELENLM KDDNQWAHPR KDYANTGYSR LSQINKGNVR NLKMAWSFAT G--VNRGHEG APLVI----- NDVMYVHTAF -P-----NNV YALDLND--N |
40_YP_0036 ---------- -S-DDLQKHL QNDKEWTHPR KDYANTGYSG LHQIQAKNIN KLKLAWTFST G--VNRGHEG SPIVL----- GSTMYLHTAF -P-----NNI YALDLNN--N |XoxF4
41_YP_5464 ---------- -A-ADQEAAM KDPNNWVHPR GQHNNQGYST LNQINKSNVK NVKAAWQFAT G--VNRGHEG SPVVI----- GNMMYVHTAF -P-----NNV YALDLND--N |___
17_WP_0122 QGATVSGRSE HPKPPSSAAP RDDGQWTMPA KNFASTRYSD LAEITPDSVK NLRVNFTFSV G--VNRGQES SPLVI----- DGTMFVLSPY -P-----NIL YALDLTK--P |
18_WP_0125 ---------- -QTPPPFVKA DAAKEWTMPA KDVASTRYSS LDQINAGNAR DLKVAFSFSL G--TDRGAEA APLVV----- GDTMYVVTPY -P-----NTL FALDLKQ--S |XoxF3
19_xoxF_1_ ---------- -QSPPSFAKA DQDGQWTMPA KDVASTRYSS LDQINAQNVK DLKVAFTFST G--TDRGVES APLVV----- GDTMYIVAPY -P-----NTL FALDLKN--D |___
29_Methyla ---------- -N-EELLRLG ADPGQWPMQA KNYEATRYSE LDQISSRTVE KLHVVWSFST G--ALRGHEG GPLVI----- DNTMYVHSPF -P-----NTV YALDLRQ-KP |
31_Methyla ---------- -N-EELLRLG ADPGQWPMQA KNYEATRYSE LDQISARTVE RMHVVWSFST G--ALRGHEG GPLVI----- GSTMYVHSPF -P-----NTI YALDLRQ-KP |
30_Methyla ---------- -N-EELLRLG SDPGQWPMQA KNYAATRYSE LDQINTRTVE RLHVAWSFST G--ALRGHEG GPLVV----- GSTMYVHSPF -P-----NTV YALDLAQ-KP |
32_NZ_JAFS ---------- -N-EELLRLG ADPGQWPMQA KNYAATRYSE LDQINARTVE RLRVAWSFST G--ALRGHEG GPLVV----- GGIMYVHSPF -P-----NTV YALDLAK-KP |
20_Methyla ---------- -N-EELVRLG ADPGQWPMAG KNYAATRYSE LDQINTRTVE RLHVAWSFCT G--ALRGHEG GPLVV----- GSTMYVHSPF -P-----NTV YALDLAR-KP |
WP_1343907 ---------- -N-DELIKLE KEPGQWVMQN KNYANTRYSE LNQINTKNVS RLRLAWSFST G--ALRGHEG GPLVV----- GTTMYVHSAY -P-----NHV YALDLTQ-KP |XoxF2
TFE68643.1 ---------- -N-DELVKLE KDPAQWVMQN KNYANTRFSE LTQINTKNVS RLRLAWSFST G--ALRGHEG GPLVV----- GSTMYVHSAY -P-----NHV YALDLTQ-KP |
WP_1344401 ---------- -N-DELVKLE KDPAQWVMQN KNYANTRFSE LTQINTKNVS RLRLAWSFST G--ALRGHEG GPLVV----- GSTMYVHSAY -P-----NHV YALDLTQ-KP |
MPANT_v1_1 ---------- -N-EELIRLS KNPGDWPMPR KNYSATAYSE LNQINTRNVG KLTQVWSFAT G--ALRGHEG SPLKI----- GDTLYVHSAF -P-----NHV YALDLTK-KP |
25_LM99741 ---------- -N-DELIKLE KEPGQWVMQN KNYANTRYSE LNQINTKNVS RLRLAWSFST G--ALRGHEG GPLVV----- GTTMYVHSAY -P-----NHV YALDLTQ-KP |
26_CP00097 ---------- -N-DELIKLE KDPAQWVMQN KNYANTRFSE LTQINTKNVS RLRLAWSFST G--ALRGHEG GPLVV----- GSTMYVHSAY -P-----NHV YALDLTQ-KP |
28_JQNX010 ---------- -N-DELIKLE KEPGQWVMQN KNYANTRYSE LNQINTKNVS HLRLAWSFST G--ALRGHEG GPLVV----- GTTMYVHSAY -P-----NHV YALDLTQ-KP |___
15_xoxF_3_ ---------- GSDEEIVRNS KNPDLWPGMG QNLALQRHSD LKDLNKDNIG NLQLAWSQST G--ALRGHEG QPVVV-DVDG KPMMFFVSAW -P-----NIV QALDLSNPDN |
16_WP_0125 ---------- GSDEEIIKNS KNPDLWPGMG QNLGLQRHSE LKDINKDNVS NLQMSWSQSS G--ALRGHEG QPVVV-DVGG KPMMFFVSAW -P-----NIV QALDLSDPDN |
35_RUP1086 ---------- -E-DEVAHRA ANPNEWAAPG RDNALTRHSP LKDINTDNVS KLQMVWSQST G--ALRGHEG QPVVIEDVGG KPMMFFVSGC -PEMSKCNIV QGLDLSDPDH |
36_WP_0200 ---------- -N-DEVDKRA ADPNQWAAPG RDNHLTRHSP LKDINTDNVG KLQMVWSQST G--ALRGHEG QPLFIEDVGG KPMLFFVSGC -PNMAQCNIA QGIDLSDPDN |
27_Methyla ---------- -N-DDVLKLT ENPKNWAAPG KDYANTRHSP LKQINTQNVK GLHMAWSFST G--VLRGHEG QPLVI----- GDRMYVVTPY -P-----NIV WALDISKGNS |XoxF1
21_Methyla ---------- -N-DDVLKLT ENPKNWAAPG KDYANTRHST LKQINTQNVK SLHMAWSFST G--TLRGHEG QPLVI----- GDRMYVVSSY -P-----NIV WALDLSK-GA |
22_NZ_JAFS ---------- -N-DDVLKLT ENPKNWAAPG KDYANTRHSS LKQINTQNVK SLHMAWSFST G--TLRGHEG QPLVV----- GDRMYVVSSY -P-----NIV WALDLSKGGS |
23_Methyla ---------- -N-EEVLKLT ENPKNWAAPG KDYGNTRHSS LKQINTQNVK SLHMAWSFST G--TLRGHEG QPLVI----- GDRMYVVSSY -P-----NIV WALDLSKGGS |
24_Methyla ---------- -N-DDVLKLT ENPKNWAAPG KDYANTRHSS LKQINTQNAK SLHMAWSFST G--TLRGHEG QPLVV----- GDRMYVVSSY -P-----NIV WALDLSKGGS |___
45_YP_0012 ---------- -N-DSAMKAA AEPGAWAMAG HDYGNTRFSP LKEINTENAG KLSLVYSFSL A--SLRSNEA SPIVI----- GNTLYVSTSW GP-----KYV YALDAA---T |
46_YP_0075 ---------- -N-ESARKAA SEPGAWAMAG HDYGNTRFSP LKEISTENAG KLSLVYSFSL A--SLRSNEA SPIVI----- GNTLYVSTSW GP-----KYV YALDAA---T |ADH's
43_YP_0030 ---------E VTEQDILNDA KTTDDVVTYG LGPQAQRFSP IKTLNRDNIK GLVPAWSFSF GGEKQRGQES QALVK----- DGVLYVTGSY -------SRL YAVDAR---T |
44_YP_0079 ---------D VSIDDILNDA KTTQDVVTAG LGGQGQRYSP LNEINKDNAA SLVPAWAMSL GGEKMRGQES QALVK----- DGIMYVTGSY -------SRV WAIDTK---T |___
                                                    *                                *
```

```
GTIL-WQDKP K----QNPAA -RAVACCDLV NRGLAYWPGD GKTPALILKT QLDGNVAALN AETGETVWKV ENSDIKVGST LTIAPYVVKD K-----VIIG SSGAELGVRG |---
GVIL-WEDKP K----QNPAA -RAVACCDVV NRGLAYWPGD GKTPSLILKT QLDGHVVALN AATGEVYWKV ENSDIKVGST LTIAPYVVKD I-----VLVG SSGAELGVRG |
TRIL-WQDKP K----QNPAA -RSVACCDLV NRGLAYWPGD SKTPPLILKT LLDGHIEALN AKTGELVWKM ENSDIKVGST LTVAPYVVKD N-----VLVG SSGAELGVRG |MxaF
GHIL-WQHSP K----QDPAA -RSVACCDLV NRGLAYWPGD DKTPALVIKT QLDGHLVALN AKTGEEFWKV ENGDIKVGQT LTQAPYVVHD L-----AIVG SSGAELGVRG |___
GKIL-WKYEP K----QDPNV -IPVMCCDTV TRGVAY--AD GK----IFLY QADTTLVALN AATGAHIWST KNGDPAKGET GTSAPQVVKD K-----VLVG ISGGEFGVRG |
GKIL-WKYEP K----QDPNV -IPVMCCDTV TRGVAY--GD GK----IFLY QADATLVALD AHTGAHIWSA KNGDPSKGET GTSAPQVVKD K-----VLVG ISGGEFGVRG |
GKIL-WKYEP K----QDPNV -IPVMCCDTV NRGVAY--AD GK----IIQY QADTTLVAVD AKTGAVVWSV KNGDPAKGET GTSAPQVIKN K-----VLVG ISGGEFGVRG |
GKIL-WKYEP K----QDPSV -IPVMCCDTV TRGVAY--GD GK----IILG QADTTLVALD AKTGQPVWSV KNGDPAKGET NTSAPQVIKD N-----VIIG ISGGEFGVRG |
SKIL-WKYEP K----QDPSV -IPVMCCDTV NRGLAY--AD GA----IILH QADTTLVSLD AKTGKVNWSV KNGDSKVGET NTATVLPVKD K-----IIVG ISGAEYGIRG |
AKIV-WKYEP K----QDPSV -IPVMCCDTV NRGLAY--AD GA----ILLH QADTTLVSLD AKSGKVNWSV KNGDPSKGET NTATVLPVKD K-----VIVG ISGGEFGVQC |
GKIL-WRYEP Q----QDPNV -IAVMCCDTV YRGLSY--AD GM----ILLG QADTTVVALD ATSGEVKWST KIGDPGIGET LTATVVPVKD K-----VLVG ISGGEYGVRG |
GKIL-WKYEP R----QDAGV -VPLMCCDTI SRGLAY--GD GK----IFLY QADTTLVALD AKTGAPVWSV KNGDPAKGAT GTSAPALIKD K-----VLVG VSGGEFGFRG |XoxF5
GKIL-WQYEP R----QDAGV -VAVMCCDVV SRGLAY--GF GK----IFLY QADATLVALD AVTGKPAWSV KNGDPAKGAT GTSAPIVVKD K-----VLVG VAGGEFGLRG |
QKIL-WKYQP K----QDPSV -IGVMCCDSV NRGVQY--AE GK----ILVH TADTSLIALD AKTGMELWKV TDDDPKKGAT GTGAPLIVKD K-----VIVG VSGGEFGVRC |
QKII-WKYEP K----QDPSV -IPVMCCDTV NRGLAY--AD GK----IFLH QADTSLVALD AKTGKELWKV VDGDPKKGET GTQAPMVVKD K-----VVIG ISGAEFGVRC |
GKIL-WRYAP R----QDASV -IAVMCCDTV SRGLAY--AE GR----LFLQ LADTTVVALD PETGRVLWSV KNGDPARGET NTATVLPVHG K-----LIVG IAGGEYGARC |
GKIL-WRYAP R----QDAGV -IAVMCCDTV SRGLAY--AD GR----LFLQ LADTTVVALD PETGRVLWSA RNGDPAKGET NTATVLPVHG K-----LIVG IAGGEYGARC |
QSVI-WEYVY APDKGTDQSQ VISVMCCDVV NRGLAY--GD GK----IFLA QGDATLVALD AKTGKIVWKV KNGDPKTGMT ATNAPLVVKD K-----VLTG ISGGEFGVRG |
QSVI-WEYTP T----MDADV TIPVMCCDTV NRGLAY--GD GK----IFLQ QSDTVLTALD AKTGKRVWSV QNGDPKLGMT NTQAPLVVKD K-----VITG ISGGEFGVRG |
QSVI-WEYTP T----MDADV TIPVMCCDTV NRGLAY--GD GK----IFLQ QSDTVLTALD AKTGKRVWSV QNGDPKLGMT NTQAPLVVKD K-----VITG ISGGEFGVRG |___
QKII-WSYFP K----QDPSV -QALLCCDNV NRGLGY--GD GK----IFLQ QNDGTLVALD AKNGNKLWDV KVVDTKTGAS NTNAPHVIKD K-----VITG CSGGEYGVRC |
QKIL-WSYFP K----QNPQT -QALLCCDTV NRGLGY--GD GK----IFLQ QNDGILVALD AKTGKLIWQT KVNDIKTGAS NTNAPHVMKD K-----VITG CSGGEYGVRC |XoxF4
QKIV-WAYFP K----QDPSV -QAVLCCDNV SRGLGY--GD GK----IFLQ QNDGVLVALD AKTGAKVWDV KVNDPKVGAT NTNAPHVIKD K-----VLTG CSGAEFGVRC |___
GAPLKWQYNP K----PEAAA -QGVACCDVV NRGPTF--AD GH----IFFN TLDGNVVSVD AATGQEAWKT KVGNIHVGET MTMAPLVARG K-----VFVG NSGGEFGVRG |
GALK-WRYDP L----PSAAS -QGEACCDVV NRGAVF--AD GK----LIYN TLDDYTIAVD AASGKEVWRT KMGDINMGET MTMAPLLMKD K-----VLVG DSGGEMGVRG |XoxF3
GALK-WRYDP K----PSRAS -QGVACCDVV NRGAVF--AD GK----LIYN TLDDYTVAVA ADTGQEVWRT KMGEINKGET MTMAPLLARD K-----VLVG DSGGEMGVRG |___
YTIL-WRYTP E----QKPSA -VTAACYDTV SRGVAY--GM GK----IFLG TLDGQLIALD AHTGDEVWKV KQGEISRGET ITGAPIVIKD M-----VIIG ISGGEFGVRG |
YTIL-WKYTP E----QKPSA -IAAACYDTV SRGVAY--GM GK----IFLG TLDGQLIALD AHTGNEVWKV KQGEIARGET ITGAPIVIKD M-----VIIG ISGGEFGVRG |
YSIL-WKYTP A----QKASA -VTEACYDTV SRGVAY--GM GK----IFLG TLDGQLIALD ARTGAEVWKV KQGEIARGET ITGAPMVIKD M-----VLVG ISGGEFGVRG |
YAIL-WKYTP E----QKPTA -VTEACYDTV NRGVAY--GM GK----IFLG ALDGELIALD AHTGAEVWKA KQGEIGRGET ITGAPIVIKD M-----VLVG ISGGEFGVRG |
YAIL-WKYTP A----QKPGA -ITEACYDTV NRGVAY--GM GK----IFLG TLDGQLIALD AHTGDEIWKV KQGEIGRGET ITGAPIVIKD M-----VLVG ISGGEFGVRG |
YAIK-WQYTP V----QNSQA -VAVACCDVV NRGLAY--AN GK----IFMV TLDGQIIALD ANTGKELWKT KHADVTKGET LTGAPLVVKD K-----ILVG VSGGEFGVRG |XoxF2
YAIK-WQYTP V----QNSQA -VAVACCDVV NRGLAY--AN GK----LFLA TLDGQIIALD ANTGKELWKT KHADVTKGET ITGAPLVVKD K-----VLVG VSGGEFGVRG |
YAIK-WQYTP V----QNSQA -VAVACCDVV NRGLAY--AN GK----LFLA TLDGQIIALD ANTGKELWKT KHADVTKGET ITGAPLVVKD K-----VLVG VSGGEFGVRG |
YAIK-WKYTP A----QNQQA -VAVACCDTV NRGLAY--GD GK----IILV TLDGQVIALD AQTGKEVWKA KNADPTKGET ITAAPLVVKD K-----VLVG VSGGEFGVRG |
YAIK-WQYTP V----QNSQA -VAVACCDVV NRGLAY--AN GK----IFMT TLDGQIIALD ANTGKELWKM KHADVTKGET ITGAPLVVKD K-----VLVG VSGGEFGVRG |
YAIK-WQYTP V----QNSQA -VAVACCDVV NRGLAY--AN GK----LFLA TLDGQIIALD ANTGKELWKA KHADVTKGET ITGAPLVVKD K-----VLVG VSGGEFGVRG |
YAIK-WQYTP V----QNSQA -VAVACCDVV NRGLAY--AN GK----IFMV TLDGQVIALD ANTGKELWKA KHADVTKGET ITGAPLVVKD K-----VLVG VSGGEFGVRG |___
PVQV-WSYNK GTD--RDLSA -VPRACCDVV NRGVNY--AD GK----VFFN TLDGFLIALD AKTGKEVWVV KHAFPEHGET VTNAPLIAKD K-----VIVG FGGDEFAARG |
PVQV-WSYNK GTD--RDVSA -VPRACCDVV NRGVNY--AD GK----LLFN TLDGFLIALD AKTGQELWVV KHAFPEHGET VTSAPLIAKD K-----VIVG FGGDEFAARG |
PKQI-WNYVK QTD--RDETA -VPRACCDTV NRGPAY--GD GK----VVFG TLDGFLIALD AQTGKEAWVV KHAWPEKGET ITNAPLIADG L-----VIIG FGGDEFAARG |
PKQI-WNYVK TTD--RDESA -VPRACCDTV NRGPAF--AD GK----LYYG TLDGFLIALD AATGKEVWVV KHAWPEKGET STSAPLIADN V-----VILG FGGDEFAARG |
YEVL-WKYAP R----QDDKA -VSTACCDTV NRGASY--AD GK----IVFN TLDGYVVCLD ANTGKELWKT KFADVNKGET STPAPIIVKD K-----VVTG YGGDEFGARG |XoxF1
YEVL-WKYVP R----QDDKA -VSTACCDTV NRGASY--AD GK----IVFN TLDGYVVCLD ANTGKELWKT KFADVNKGET STPAPIIAKD K-----VFTG YGGDEFGARG |
YEVL-WKYVP R----QDDKA -VSTACCDTV NRGASY--AD GK----VVFN TLDGYVVCLD ANTGKELWKT KFADLNKGET STPAPIIVKD K-----VFTG YGGDEFGARG |
YEVL-WKYVP K----QDDKA -VSTACCDTV NRGASY--AD GK----VVFN TLDGYVVCLD ANTGKELWKT KFADLDKGET STPAPIIVRD K-----VFTG YGGDEFGARG |
YEVL-WKYVP R----QDDKA -VSTACCDTV NRGASY--AD GK----VVFN TLDGYVVCLD ANTGKELWKT KFAEVNKGET STPAPIIAKD K-----VFTG YGGDEFGARG |___
GARK-WTYEP E----IPDDT -LQYACCDVN SRGITY--AD GK----LFVG RLDGKLTALD AATGKELWTA KVVDYKQGSV ITSPPLVVRD K-----VITG FGGGEYGVRG |
GARK-WTYEP D----IPDDV -LQYACCDVN NRGVTY--AD GK----LFVG RLDGKLTALD AVTGKELWTS KVVDYKQGSV ITSPPLVVRD K-----VITG FGGGEYGVRG |ADH's
GEKK-WEYNA R----LPEGI ---LPCCDVV NRGAAL--YK DK----VYFG TLDAKLVALD QKTGKVVWRK DIDDFKSGYS YTAAPMIVKG K-----IITG VSGGEFGVVG |
GHEI-WEYDA R----LPEGI ---LPCCDVV NRGAAL--IG DK----VIFG TLDAKLVALN QKTGKVVWMK EIDDFKGGYS YTAPPLIVPT KTFGPLIITG VSGGEFGIVG |___
     *                     * *    **                     *           *   *          *    *                  *   * *
```

```
YLTAYDVKTG EQVWRAYATG PDKDLLLASD FNIKNPHYG- ---------- ---------- --------QK GLGTGTW--- --EGDAWKIG GGTNWGWYAY DPGTNLIYFG |---
YVTGYDVKTG EQKWRAYATG PDSDLLLADD FNIKNPHYG- ---------- ---------- --------QK GLGTSTW--- --EGDAWKIG GGTNWGWYAF DPDTNMTYFG |
YVTAYDVHDG EQKWRAYATG PDKDLLLAAD FNIHNPQYG- ---------- ---------- --------QK GLGLDTW--- --EGDAWKIG GGTNWGWYAF DPKTNLIYFG |MxaF
HVTAYNVKTG EQAWRYYATG PDEEIGLADD FNSANPHYG- ---------- ---------- --------QK GLGTATW--- --EGDAWKIG GGTNWGWYAY DPQANLIYYG |___
SLTAYDIKTG KKVWRGYSMG PDSDTLIDPE ---KTTHLG- ---------- ---------- -----KPVGK DSGINTW--- --QGDQWKIG GGTTWGWYSY DPDLNLVYYG |
HITAYDIKTG KKAWRGYSVG PDSDTLIDPE ---NTTHLG- ---------- ---------- -----KPVGK DSGINTW--- --QGDQWKTG GGTTWGWYSY DPELNLVYYG |
HVTAYDLKTG KKVWRGYSVG TDADTLIDPE ---KTTYLG- ---------- ---------- -----KPVGK ESSTSSW--- --KGDQWKIG GGATWGWFSY DPDLNLIYYG |
SVTAYDLKTG KKVWRGYSMG PDSDTLIDPE ---KTTALG- ---------- ---------- -----KPVGA NSGTNTW--- --EGDQWKIG GGTTWGWISY DPELNLVYYG |
HMTAYDAKTG KRVWRAYSVG PDDEMLVDPE ---KTTSLG- ---------- ---------- -----KPIGK DSSLKTW--- --EGDQWKTG GGATWGWYSY DPKLDLFYYG |
HVTAYDLKSG KKVWRGYSIG PDDQLIVDPE ---KTTSLG- ---------- ---------- -----KPIGK DSSLKTW--- --EGDQWKTG GGCTWGWFSY DPKLDLMYYG |
RMTALNLTDG SEAWKAWSTG PDEELLVDPE ---TTTHLG- ---------- ---------- -----KPIGA DSSLNSW--- --EGDQWQIG GGTIWGWFSY DPDLNLVYYG |
YLTAYDVKTG AKLWRAYSVG PDADTLIDPE ---KTTELG- ---------- ---------- -----KPVGK ESSLASW--- --QGDQWKIG GGAPWGRISY DAAENLIYYG |XoxF5
YLTAYDAATG AKIWRAYSTG PDSETLIDPV ---TTRELG- ---------- ---------- -----RSVGA EASLKSW--- --SGDQWALG GGAPWGRISY DPGLNLVYYG |
YITAYDLNSG YKVWRAYAMG PDEDVLVDPE ---KTLVLG- ---------- ---------- -----KPAGK DSSLSTW--- --NGDQWKIG GGPTWGYMAY DPELNLIYYG |
HMTAYDINSG KKVWRAYSMG PDSDILVDPE ---KTMMLG- ---------- ---------- -----KPVGK DSSLKTW--- --NGDQWKIG GGSTWGYMAY DPDLNLMYYG |
HVTAYDQASG KRLWRAYATG PDADMLVDPE ---KTTELG- ---------- ---------- -----HPIGR DSSLKSW--- --DGDQWRIG GGCSWGWFSY DPALNLVYYG |
HVTAYDQASG QRLWRAYATG PDADMLVDPE ---KTTELG- ---------- ---------- -----RPIGR DSSLRSW--- --EGDQWRIG GGCSWGWFSY DPDLNLVYYG |
FLAAYNIKDG SLVWKKYSMG PDDEVGLDPE ---HTMTWT- ---------- ---------- -DGKMAPVGK DSSLKTW--- --QGDQWKIG GGTTWGWYSY DPDLNLVYYG |
FLAAYNIRTG QLDWKGYSMG PDGDTLIDPK ---KTTAWK- ---------- ---------- -DGKVQPVGP NSSTSTW--- --EGDQWKIG GGTTWGWYSY DSELNLVYYG |
FLAAYNIRTG QLDWKGYSMG PDGDTLIDPK ---KTTAWK- ---------- ---------- -DGKVQPVGP NSSLSTW--- --EGDQWKIG GGTTWGWYSY DSELNLVYYG |___
YISAYNIKDG SLAWRAYSTG PDSETLIGDG FNKDNPHYSA LSVYEDVNGG NKEGGSFKAL TKDQMKFPEK ELGTRTWLKP QAIKNGYEHG GGPTWGWYSF DPALNLMYYG |
FIAAYNIKDG SLAWKAYSTG PDSETLIGAG FNEANPHYSA LSTYQDVNGG NKEDGSFKAL TKEQLKFPEA DLGVRTWLKP QANQDGWQHG GGPVWGYFSY DPKLNLLYYG |XoxF4
FLVAYNIKDG SQVWKAYSTG PDSEVLIGKD FNKDNPHYSA LSVYEDINGG NKEGGSFKAL PKEKLKFPET DLGVKTWLKP QAVKDGWQHG GGSTWGWWPY DHRTNLVYYG |___
WIQALDQQSG KVAWKAFNTG PDKDVLIGPE F---KPFYD- ---------- ---------- -----MDKGQ DLGVKTW--- --PPQAWEQG GGTVWGWLSY DPDLNLLYHG |
WGAALDVKTG KIVWRAYSTG PDSDVLIGAA F---KPFYD- ---------- ---------- -----SDKGK DLGVTTW--- --PADMWRQG GGSVWGWASY DPELNLVYYG |XoxF3
WAAALDVKTG KILWRAYNTG PDSDVLIGSE F---KPFYD- ---------- ---------- -----SEKGK DLGVTTW--- --PADMWRHG GGSVWGWASY DPDLNLIYYG |__
RINAYDINTG KPRWVAYSQG PDDDILLEGD FNKECPRHG- ---------- ---------- --------GE GEGTRSW--- --PGEQWKLG GGTTWGWFSY DSDLDLFHYG |
RINAYDIQTG KPRWVAYSQG PDDDILLEGD FNKECPRHG- ---------- ---------- --------GE GEGTRSW--- --PGEQWKLG GGTTWGWFSY DPDLDLFHYG |
RVNAYDINTG KPRWVAYSQG PDDDILLEGD FNKECPRHG- ---------- ---------- --------GD GEGTRSW--- --PGEQWKLG GGTTWGWFSY DPDLDLFHYG |
RVNAYDANTG KPRWVAYSQG PDDDVLLEGN FNKECPRHG- ---------- ---------- --------GE GEGTRSW--- --PGEQWKLG GGTTWGWFSY DPDLDLFHYG |
RVNAYDINTG KARWVAYSQG PDDDILLERD FNKECPRHG- ---------- ---------- --------GP GEGTRSW--- --PGEQWKLG GGTTWGWFSY DPGLDLFYYG |
RVGAYDINTG NRVWLAYSQG PDEEVLLDSD FNKEFPKHG- ---------- ---------- --------GP GDGTKTW--- --PGEQWKLG GGTTWGWYSY DPALDLFYYG |XoxF2
RVGAYDINTG NRVWLAYSQG PDEEVLIDSD FNKEFPQHG- ---------- ---------- --------GP GDGTKTW--- --PGEQWKLG GGTTWGWYSY DPALDLFYYG |
RVGAYDINTG NRVWLAYSQG PDEEVLIDSD FNKEFPQHG- ---------- ---------- --------GP GDGTKTW--- --PGEQWKLG GGTTWGWYSY DPALDLFYYG |
RMTAYNLANG ERAWLGYNQG PDEDILLDPD FNKECPHHG- ---------- ---------- --------GP GDGVKSW--- --PDEQWKIG GSTVWGWFSY DPELDLVYYG |
RVGAYDINTG NRVWLAYSQG PDEEVLLDSD FNKEFPQHG- ---------- ---------- --------GP GDGTKTW--- --PGEQWKLG GGTTWGWYSY DPALDLFYYG |
RVGAYDINTG NRVWLAYSQG PDEEVLLDSD FNKEFPQHG- ---------- ---------- --------GP GDGTKTW--- --PGEQWKLG GGTTWGWYSY DPALDLFYYG |
RVGAYDINTG NRVWLAYSQG PDEEVLIDSD FNKEFPQHG- ---------- ---------- --------GP GDGTKTW--- --PGEQWKLG GGTTWGWYSY DPALDLFYYG |___
RLEAYDLATG KLAWSFESNG TDKDVGLTPD TNKAHPEHG- ---------- ---------- ------VYGS DIGLSSY--- --PGDEWKRG GGSPWAWYSY DPELGLVYAS |
RLEAYDLATG DLAWRCQSNG TDKDVCLTPD TNKAHPEHG- ---------- ---------- ------TYGH DIGLSSY--- --PGDEWKRG GGSPWAWYSY DPELGLVYAS |
RVNAYDLKTG KQVWKCYSTG SDKDVCLTAE TNKANPHYG- ---------- ---------- ------TAGK DLGIHTH--- --VGDDWKIG GGSAWGWFSY DPELKMVYYS |
RVTAYDLKDG KKVWECHSTG SDKDVCLTPE TNKANPHYG- ---------- ---------- ------TAGK DLGIHTH--- --VGEDYKIG GGAAWAWFSY DPELKLVYYS |
RFAAFDLNSG KMVWQAYSNG PDSDVLLGPD FNSKHPEYG- ---------- ---------- ------QAGQ DLGVKTY--- --PDEEWKRG GGCAWGWYSY DPKLDLIYYN |XoxF1
RFAAFDLNSG KMVWQAYSNG PDSDVLLGPD FNSKHPEYG- ---------- ---------- ------QAGQ DLGVKTY--- --PDEEWKRG GGCAWGWYSY DPKLDLVYYN |
RFAAFDLNSG KMVWQAYSNG PDSDVLLGPD FNSKHPEYG- ---------- ---------- ------QAGQ DLGVKTY--- --PDEEWKRG GGCAWGWYSY DPKLDLVYYN |
RFAAFDLNSG KMVWQAYSTG PDSDVLLGSD FNSKHPEYG- ---------- ---------- ------QAGQ DLGVKSY--- --PDEEWKRG GGCAWGWYSY DPKLDLVYYN |
RFAAFDLNSG KMVWQAYSNG PDSDVLLGPD FNSKHPEFG- ---------- ---------- ------QAGQ DLGVKTY--- --PDEEWKRG GGCAWGWYSY DPKLDLVYYN |___
SLQAYDINSG KMVWQTYTVP APGE------ ---------- ---------- ---------- ---------- -PGSETW--- --KGDTGLHG GGAAWLVGSY DPKSDTVYWG |
SLQAFDINSG KALWQTYTVP APGE------ ---------- ---------- ---------- ---------- -PGSETW--- --KGDTGLHG GGAAWLVGSY DPKTDTVYWG |ADH's
RVEARDAETG EVVWKR---- PVIEGHMGEL NGKPSTMTG- ---------- ---------- ---------- -KANESW--- --PGDTWKFG GGATWLGGTY DPELNLIYFG |
RIDARKADNG ELVWTRPTIE GHMGTLNGS- ---ESTMTG- ---------- ---------- ---------- -KKNETW--- --KGDLWKTG GGATWLGGTY DAETNTIFVG |___
         *    *                                                                                  * *   *      *
```

```
TGNPAPWNET MRPG------ -------DNK WTMTIFGRDA DTGEAKFGYQ KTPHDEWDYA GVNVMMLSEQ KD---KDGKA RKLLTHPDRN GIVYTLDRTD GALVSANKLD |---
TGNPAPWNET MRPG------ -------DNK WTMTIFGRDV DTGKAHFGYQ KTPHDEWDFA GVNVMMLSDQ KD---KDGKL RKLLTHPDRN GIVYTLDRTN GDLVSANKID |
TGNPSPWNET MRPG------ -------DNK WTMTIFGRDA DTGLAKFGYQ KTPHDEWDYA GVNVMMLSEQ KD---KDGQV RSLLTHPDRN GIIYTLDRTN GDLVSANKID |MxaF
SGNPAPWNET MRPG------ -------DNK WTMTITARDA DTGKMKFGYQ KTPHDEWDFA GVNVIMLSEQ TD---KEGKK RKLLTHPDRN GIVYTLDREN GDLISADKLD |___
SGNPSTWNPK QRPG------ -------DNR WSMAIWARDA DTGVAKWIYQ MTPHDEWDYD GINEMIL--- -----VGQEA RKVLVHFDRN GFGYTLDRVS GELLVAEKFD |
SGNPSTWNPK QRPG------ -------DNR WSMTIWARDA DDGKVKWVYQ MTPHDQWDYD GINEMIL--- -----VNQEA RKVLVHFDRN GFAYTLDRVT GEPLVAEKFD |
SGNPSTWNPV QRPG------ -------DNR WSMTIWARDA DTGVAKWVYQ LTPHDQWDYD SINESIL--- -----VGQEA RKVLVHFDRN GFGYTIDRVT GEPLVAEKFD |
SGNPSTWNPV QRPG------ -------DNR WSMSVWARDA DSGKVKWIYQ MTPHDEWDYD GINEMVLTEQ K----IGGKE RKLLTHFDRN GFGYTLDRVT GELLVAEKYD |
TANPSTWNPK QRPG------ -------DNK WTMAIFARNP DTGQAKWIYQ MTPHDEWDYD GINEMILTDQ K----VDGKE RPLLTHFDRN GFAYTLDRAN GEVLVAEKFD |
SGNPSTWNPK QRPG------ -------DNK WSMTIWARNP DTGMAKWVYQ MTPHDEWDFD GINEMILTDQ K----FDGKD RPLLTHFDRN GFGYTLDRAT GEVLVAEKFD |
TGNPSTWNPS QRPG------ -------DNK WSMTIMARDA DTGMAKWFYQ MTPHDEWDYD GVNEMILTNQ T----VDGQE RKLLTHFDRN GLAYTMDRET GELLVAEKYD |
TGNPSTWNPT QRPG------ -------DNK WSMAIFARDV DTGVARWVYQ MTPHDEWDYD GVNEMILTDA S-------GG RKVLTHFDRN GFAYVLDRAD GDLISADKYD |XoxF5
TGNPSTWNPA QRPG------ -------DNK WAMTILARNA DTGEAAWLYQ MIPHDQWDYD GVNEMILGEA A----IDGRA RKTLTHFDRN GFVYVLDRAD GELLSADKYD |
TGNPSTWNPV QRAGPDRKPI -------DQK WAMTMFARDV DTGIARWAYQ MTPFDQWDYD GINEPILADL E----INGFP RKTVTHFDRN GFAYTWDRAT GDLLVAEKFD |
TGNPSTWNPS QRAGPDGKPI -------DQL YSMTMFARDV DTGVAKWAYQ MTPFDEWDYD GINEPILAEL D----FDGTK RAIATHFDRN GFGYTWDRAT GELLVAEKYD |
TGNPATWNPA QRPG------ -------DNR WTMAIIARDP DTGLARWVYQ MTPHDQWDYD GVNEMILTEQ V----IDGRP RRVLTHFDRN GFGYTIDRAT GDLLVAEKFD |
TGNPATWNPA QRPG------ -------DNR WTMAIIARDP DTGMARWVYQ MTPHDQWDYD GVNEMILTEQ V----IDGRP RRVLTHFDRN GFGYTLDRAT GEVLVAEKFD |
SGNPSTWNPV QRPG------ -------DNK WSMTIWARDV DTGEAKWVYQ MTPHDEWDYD GINEMMLIDQ EMTAKDGSKH SKLLTHFDRN GFGYTLDRVT GELLVAEKFD |
SGNPSTWNPV QRPG------ -------DNK WSMSLWARDA DTGAVKWVYQ MTPHDEWDYD GINETVLVDQ K----VKGKM RKLIVHFDRN GFGYTLDRVT GELMVAEKFD |
SGNPSTWNPV QRPG------ -------DNK WSMSLWARDA DTGEVKWVYQ MTPHDEWDFD GINETVLVDQ K----VKGKM RKLIVHFDRN GFGYTLDRVT GELMVAEKFD |___
TGNPGTWNPD VRPG------ -------DNK WSMTIFARDV DTGMARWGYQ MTPHDEWDYD GMNEIVL--- -----WEAKG KKLATHFDRN GFGYTFDRTN GTLLVAEKMN |
TGNPSVWNAD VRPG------ -------DNK WAMTIFARDL DTGMAKWGYQ MTPHDEWDYD GINEVIL--- -----WDANN KKLATHFDRN GFGYTFDRTN GSLLVVAKMH |XoxF4
TGNPSVWNPD VRPG------ -------DNK WSMTIFARDV DTGIAKWGYQ MTPHDEWDYD GINEVIL--- -----FDKGG KTYAWHHDRN GFAYTFDAHT GSIIAAEKVH |___
TGNPGPWNAD QRPG------ -------DNK WTSGIFARDA ETGQARWFYQ WSPHDLYDWD GINEQILLDM T----WKGQP RKVLVRPERN GYVYILDRTT GEVLSAKPF- |
VSNPGPWNSS MRPG------ -------DNK WTSGIFARDA DTGQARWFYQ YSPHDLYDYD GVNEEILLDM P----WKGQP RKVLVHPDRN GYVYVLDRVN GEVLSAEPF- |XoxF3
VSNPGPWNST IRPG------ -------DNK WTSGIFARDA DTGQARWFYQ YSPHDLYDYD GVNEEILLDL T----WKGQP RKVLVHPDRN GYVYVIDRTN GEVLSAEPF- |___
TGNPGSWNAE ERKGG----- -------DNK WSCTLWGRRP TTGKARWAFQ MTPWDSWGYD GTNELILVDL P----IGNAK RKCAVHFDKN GFVYTLDRGT GEVLSAVPF- |
TGNPGSWNTE VRKGG----- -------DNK WSCTLWGRRP GTGKARWAFQ MTPWDSWGYD GTNELILVDL P----IGNAK RKCAVHFDKN GFAYTLDRGT GEVLSAVPF- |
SGNPGSWNPD ERKGG----- -------DNK WSCTLWGRRP GTGKARWAFQ MTPWDSWGYD GTSELILIDL P----IGKTM RKCAVHFDKN GFAYTLDRGT GEVLLATPF- |
SGNPGSWNPD ERKGG----- -------DNK WACTLWGRRP ATGMARWAYQ MTPWDSWGYD GTSELVLVDL R----IGSTL RKCAVHFDKN GYAYTVDRGT GELLSAAPF- |
SGNPGSWNPD ERKGG----- -------DNK WACTLWGRRP ATGMARWAYQ MTPWDSWGYD GTSELVLVDL R----IGEGV RRCAVHFDKN GFAYTLDRAT GEVLSAAPF- |
TGNPGTWNAE QRKGG----- -------DNK WSCTIFARRP DTGKARWAYQ MTPWDAWDYD GINEMILPDL T----VKGKK TPCLVHFDRN GFGYVLDRRT GQLIEAQPF- |XoxF2
TGNPGTWNAE QRKGG----- -------DNK WSCSIIARRP DTGKARWAYQ MTPWDSWDYD GINEMILPDL T----VKGKK TPCLVHFDRN GFGYVLDRRT GQLIEAQPF- |
TGNPGTWNAE QRKGG----- -------DNK WSCSIIARRP DTGKARWAYQ MTPWDSWDYD GINEMILPDL T----VKGKK TPCLVHFDRN GFGYVLDRRT GQLIEAQPF- |
TSNPGTWNPD QRKGG----- -------DNK WSCSIIARHP DTGKARWAYQ MTPWDHWDYD GINENVLVDL N----FGGKK VKALVHFDRN GFAYTLDRET GKVLVAEPF- |
TSNPGTWNAE QRKGG----- -------DNK WSCTIFARRP DTGKARWAYQ MTPWDSWDYD GVNEMILPDL T----VKGKK TPCLVHFDRN GFGYVLDRRT GQLIEAQPF- |
TGNPGTWNAE QRKGG----- -------DNK WSCSIIARRP DTGKARWAYQ MTPWDAWDYD GINEMILPDL T----VKGKK TPCLVHFDRN GFGYVLDRRT GQLIEAHPF- |
TGNPGTWNAE QRKGG----- -------DNK WSCTIFARRP DTGKARWAYQ MTPWDAWDYD GINEMILPDL T----VKGKK TPCLVHFDRN GFAYVLDRRT GQLIEAQPF- |___
TGNPGNWSPT TRCGADTQEE CNSG-KWDNK WSMTIFARKV DTGEVVWAYQ MTPFDQWDYD GVNENILVDF PN---VDGKP VKGLVHFDRN GFAYVLDRTD GNLLRANKF- |
TGNPGNWSPT TRCGADTDAE CNSG-KWDNK WSMTIFARKV DTGEVVWAYQ MTPFDQWDYD GVNENILVDM PN---VDGKP VKALVHFDRN GFAYVLDRTD GNLLRAHKF- |
TGNPGLWSPA YRCAKKTHEE CNTG-EFDNK WSMTIFGRKV DTGEAVFAYQ MTPFDQWDYD GVNENILVDM N----VDGKD RKTLVHFDRN GFNYVLDRTD GTLLRANKY- |
TGNPGLWSPA YRCSKKTHEE CNTG-EFDNK WSMTIFARKV DTGEAVWAYQ MTPFDQWDYD GVNENILVDM D----VDGKK RKALVHFDRN GFAYVLDRAD GTLLRAHKY- |
TGNPGLWSPS YRTEAKTHEE ANEPWKWDNK WSMTIFARKP DTGEAVWGYQ MTPFDQWDYD GINEDVLVDI T----VDGSK KPCLVHFDRN GFCYVLNRTD GTIIRANKF- |XoxF1
TGNPGLWSPS YRTEAKTHEE ANEPWKWDNK WSMTIFARKP DTGEAVWGYQ MTPFDQWDYD GINEDVLVDI N----VDGAK KPCLVHFDRN GFCYVLNRTD GTLIRANKF- |
TGNPGLWSPS YRTEAKTHEE ANEPWKWDNK WSMTVFARKP DTGEAVWGYQ LTPFDQWDYD AINEDVLVDI T----VDGAK KPCLVHFDRN GFAYVLSRTD GTLIRANKF- |
TGNPGLWSPS YRTEAKTFEE ANEPWKWDNK WSMTVFARKP DTGAAVWGYQ LTPFDEWDYD AINEDVLVDI N----VDGAK KPCLVHFDRN GFCYVLNRTD GTIIRANKF- |
TGNPGLWSPS YRTEAKTHEE ANEPWKWDNK WSMTVFARKP DTGEAVWGYQ MTPFDQWDYD GINEDVLVDI N----VDGVK KPCLVHFDRN GFCYVLDRTD GTIIRANKF- |___
TSNPGPWNTA VRSTGNGDFG -----KLTNL YTASTLALDP NTGKIKWHIQ TTPADAWDYD GVNEAVLADL N----VGGAA VPTLMKADRN GFFFVANRET GKVISAEKY- |
TSNPGPWNTA VRSTGNGDFG -----KLTNL YTASTLAIDP NNGAIKWHIQ GTPADAWDYD GVNEALLADL K----IGGAS VPTLMKADRN GFFFVANRET GKVISAEKY- |ADH’s
TGNPGPWNSA LRPG------ -------DNK WSASRLAINP DNGEIVWGFQ TTPHDGWDYD GVNEFVPFDM Q----KDGKT VKAGATADRN GFFYVLDRTN GAFLSATPFV |
TGNPAPWNSH MRPG------ -------DNR WSCSRLAIDP ETGNIKWGFQ TTPNDGWDFD GVNEVVS--- -----FDLDG TKAVATADRN GFFYVLNREN GKFIRGFPFV |___
  **  *     *                                 *      *   * *                                     * *          *
```

```
DTVNVFKSVD LKT-----GQ PVRDPEYGTR M------DHL AKDI--CPSA MGYHNQGHDS YDPKR-ELFF MGINHICMDW EPFMLPYR-A GQFFVGATLN MYPG------ |---
DTVNVFKQVD LKS-----GT PVRDPEFGTR M------DHL AREV--CPSA MGYHNQGHDS YDANK-QLFY MGINHICMDW EPFMLPYR-A GQFFVGATLN MYPG------ |
DTVNEFKSVD MKT-----GQ PIRDPEYGTR M------DHL AKDI--CPSA MGYHNQGHDS YDPQK-QLFF MGVNHICMDW EPFMLPYR-A GQFFVGATLN MYPG------ |MxaF
DTVNVFKHVD LKS-----GL PVRDPEFGTR M------DHK GTEI--CPSA MGYHNQGHDS YDPTK-QLFF MGINHICMDW EPFMLPYR-A GQFFVGATLW MYPG------ |___
PAVNWATKVD MDKNSKTYGR PLVVPAFSTE HN-----GED VNSQGIMPAA LGVKDQQPAA YSPLT-GLFY IPANHVAMDY EPFRVSYT-A GQPYIGATLS MYP------- |
PAVNWATKVD LDKNSKTYGR PLVVKEFSTE SN-----GED VNSQGIFPAA LGVKDQQPAA YSPIT-NLFY VPANHVAMDY EPFRVSYT-A GQPFVGATLS MYP------- |
PAVNWATKVD LDKNSKTYGR PLVVKEFSTE AN-----GED VNTQGVMPAA LGVKDQQPAA YSPLT-NLFY IPANHVAMDY EPFRVSYA-A GQPYVGATLS MYP------- |
PAVNWATKVD MDKSSKSYGR PLVVPQFSTA HG-----GED VNTKGICPAA LGSKDEQPSA YSPLT-ELHY VPTNHVCMDY EPFRVSYT-A GQPYVGATLS MYP------- |
PVVNWASKVD LDKGSKNYGR PLVVSKYSTD QN-----GED VNSKGICPAA LGTKDQQPAA FSPKT-QLFY VPTNHVCMDY EPFKVTYT-P GQPYVGATLS MYPA------ |
PVVNWATKVD LDKGSKTYGR PLVVSKYSTE QN-----GED VNSKGICPAA LGTKDQQPAA FSPKT-GLFY VPTNHVCMDY EPFRVTYT-P GQPYVGATLS MYPA------ |
PVVNWTTGVD MDPNSETYGR PAVVAEYSTA QN-----GED ENTTGVCPAA LGTKDQQPAA FSPKT-NLFY VPTNHVCMDY EPFRVAYT-A GQPYVGATLS MYPA------ |
PAVNWASRID MDKSSKSYGR PIVDGAYSTQ QN-----GEE TVTHGVCPST VGAKNANPAA YSALT-GLFY APMIRLCMDY EPFQITYA-A GQPYTGADTA VYPE------ |XoxF5
PAVNWASRVD LGKSSKAYGR PVLDNAYSTE AN-----GEE TVTHNICPAT VGAKNANPAA YSEQT-GLFY APMIRLCMDY EPFHIAYA-Q GQPYTGADTA VYPQ------ |
PAANWATSIV MDKNDPHYGR PAVVKKYAPF ET-----GTD ETTKGICPSS LGSKNQQPAS YSLAT-GLFY VPTTRMCMDY EPFKVSYT-V GQPYVGATLT MYPV------ |
PAVNWATGVD MDKSSKNYGR PIRVAKYSTF KDGK---GAD VNTKGVCPAA LGSKDQQPAS FSKLT-GLFY VPTNHVCMDY EPFKVSYT-A GQPYVGATLS MFPA------ |
PSVNWARRID LDRASPTYGR PVVDPRFAPG EA-----DEE EVTKNICPGA LGAKNQQPAA YSPVT-RLFY VPTNHMCMDY EAFRVSYT-P GQPYVGATLT LHP------- |
PSVNWARRID LDRASPGYGR PVLDTRYAPG EA-----DEE EVTKNICPGA LGAKNQQPAA YSPVT-RLFY VPTNHMCMDY EAFRVSYT-P GQPYVGATLT LHP------- |
KAVNWATHVD MKT-----GR PQVNPKYSTQ HG-----GQD VDTKGICPSA MGAKNEPPVT YSPRT-KLIY IPGNHTCMNY EPFEVEYT-A GQPYVGATLN IFPARANVKT |
KSVNWATHVD MHT-----GR PQVVPEMSTE QN-----GED VNTVGVCPAA LGSKNQQPVS FSPKT-GLFY ISGNHLCMEY EPFEVSYT-A GQPYVGATLT MQPAGADVMT |
KSVNWATHVD MHT-----GR PQVVPEMSTE QN-----GED VNTVGVCPAA LGSKNQQPVS FSPKT-GLFY ISGNHLCMEY EPFEVSYT-A GQPYVGATLT MQPAGADVMT |___
PFVNWATKVD LKS-----GV PVKDPKFSTH Q------DYN A--KGICPSA LGVKDEQPSA FSPKT-GMFY VPLNHVCMTY EPVESKYI-A GQPWVGATLT MFAG------ |
PFVNWATSVD LST-----GI PQKDPKYSAH Q------DTT TKNV--CPAA LGVKDQQPAA YSPKT-KLFY VPLNHLCMDY DVVEAKYT-A GQPWVGAGLT MHPG------ |XoxF4
PFVNWATHVD KAT-----GI PHKDGKYSTH Q------DYN T--KGICPAA LGTKDQQPAA YSPRT-GLVY SPLNHVCMTY EPVESKYI-A GQPWVGATLT MFAG------ |___
HAITTTTGVD LKT-----GR LQYIPEKKPQ M------GKV MRDI--CPNA SGAKDWSPSA YSPRT-GLIY LPHTNLCMDE EHMQANYI-A GTPYLGSETR MKAG------ |
QKITSSTGVD LKT-----GR LQYVAAKQPK L------GET VRDI--CPAP PGAKDWQPSA FSNKT-KLLY LPHNNLCFDT EEEDVGYI-A GTPYVGASIH MHAG------ |XoxF3
QAITSSTGVD LKT-----GR LQYVEAKRPK T------GET VRDI--CPAP PGAKDWQPTA FSHKT-GLLY IPHNNLCFDT EELDVGYI-E GTPYVGANIH MKAG------ |___
VYLNWAKGIN RGD-----DR PIEVPEKRAK L------GID V--KRVCPSS MGGKNQQPAA YDPET-HLFY VGTNNLCMDI QDMEARYN-P GSPYVGADLL IYSGH----- |
VYENWAKGIN RGD-----DR PIEVPEKRAK L------GVD V--KGVCPSS MGGKNQQPAA YDPET-HLFY VGTNNLCMDI QAMEAKYN-P GTPYVGADLL IYSGH----- |
VYVNWAKGIN RGD-----DR PIEVPEKRTK A------GIE V--KGVCPSA MGGKNQQPAA YDPQT-HLLY VGTNNLCMDI EPVEARYI-P GSPYIGADLI LYSGH----- |
VYVNWAKGIS RGD-----DR PIEVPEKRAK A------GVD I--KGVCPSA MGGKNQQPAA YDPET-HLFY VGTNNLCMDI LATEVRYI-S GSPYIGADLK IYSGH----- |
VYVNWAKGIN RGD-----DR PIEVPEKRAK A------GID I--KGVCPSA MGGKNQQPAA YAPQT-HLFY LGTNNLCMDI EATEARYV-L GLPYIGANLT IYSGH----- |
VYVNWAKEIS KEN-----DR PVEIPEKRTK Q------GVD T--KGICPNS MGGKDQQPAA FSPQT-GLFY VPTNNMCMNY EGVEATYT-A GAPYVGANVL MYSGH----- |XoxF2
VYENWAKEIS KEN-----DR PVEIPEKRTK Q------GVD T--KGICPNS MGGKDQQPAA FNPQT-GLFY VPTNNMCMNY EGVEATYT-A GAPYVGANVL MYSGH----- |
VYENWAKEIS KEN-----DR PVEIPEKRTK Q------GVD T--KGICPNS MGGKDQQPAA FNPQT-GLFY VPTNNMCMNY EGVEATYT-A GAPYVGANVL MYSGH----- |
VYVNWAKGID KNT-----DR PIEVPEKLTK Q------GVD T--KNICPCA MGGKDQQPSA YSPQT-GLLY AGTNNMCMNY EGVEVKYA-A GAPYVGANVL MFSGH----- |
VYVNWAKEIS KEN-----DR PVEIPEKRTK Q------GVD --TKGICPNS MGGKDQQPAA FSPQT-GLFY VPTNNMCMNY EGVEATYT-A GAPYVGANVL MYSGH----- |
VYENWAKEIS KEN-----DR PVEIPEKRTK Q------GVD T--KGICPNS MGGKDQQPAA YNPQT-GLFY VPTNNMCMNY EGVEATYT-A GAPYVGANVL MYSGH----- |
VYENWAKEIS KEN-----DR PVEIPEKRTK Q------GVD T--KGICPNS MGGKDQQPAA FSPQT-GLFY VPTNNMCMNY EGVEATYT-A GAPYVGANVL MYSGH----- |___
VTVNWAEKVD LKT-----GR PVKVPEHSPF KI-----GVN TQA---CPSA MGGKDQQPAS VDPKDPTNFY VPTNNWCMED EPQARTHTQQ GTVYVFANVY MYPEK----- |
VTVNWAEKVD LKT-----GR PVKVPEHSPF KI-----GVN TQA---CPSA MGGKDQQPAS VDPKDPTNFY VPTNNWCMED EPQARTHTQQ GTVYVFANVY MYPEK----- |
VTVNWAEKID LKT-----GR PVKVREHSPL ER-----GRN VNA---CPSA MGGKDQQPCS VDPAEPNKFY CPTNNWCMEL EPQERSHTQQ GTVYVFANVF MFPEK----- |
VTVDWAEKVD LKT-----GR PVKVREHSPL ER-----GRN VSA---CPSA MGGKDQQPCA VDPAEPTKFY CPTNNWCMEL EPQERTHTQQ GTVYVFANVF MYPEK----- |
VTVNWAEKID MKT-----GR PVKVKEHSPF EV-----GKA VQA---YPSA MGGKDQQPVA VDPKEPNVFY APTNNWGMTL EPMERAHTNQ GSVYVFANVL MKPEK----- |XoxF1
VTVNWAEKID FKT-----GR PVKVKEHSPF EV-----GKA VQA---YPSA QGGKDQQPVA VDPKEANVFY APTNNWGMTL EPQERAHTNQ GSVYVFANVL MKAEK----- |
VTVNWAEKID MKT-----GR PVKVKEHSPF EV-----GKA VQA---YPSA QGGKDQQPVA VDPKEANVFY APTNNWGMTL EPMERAHTNQ GSVYVFANVL MKPEK----- |
VTANWAEKID MKT-----GR PVKVKEHSPF EV-----GKA VQA---YPSA QGGKDQQPVA VDPKEANVFY APTNNWGMTL EPMERAHTNQ GSVYVFANVL MKPEK----- |
VTVNWAEKID MKT-----GR PVKVKEHSPF EV-----GKA VQA---YPSA QGGKDQQPVA VDPKEANVFY APTNNWGMTL EPMERAHTNQ GSVYVFANVL MKAEK----- |___
VFANWAQKWD TST-----LR AVEDPDKRPG P------GHP VKDV--CPNL IGGKNWQPMS YSPDT-GLVY IPANNVCMDW SVGDVSYK-R GVFYLGAEFP TKEG------ |
VFANWAQKWD IAT-----SR AVEDPDKRPG P------GHP AKDI--CPNL IGGKNWQPMS YSPDT-GLVY IPANNVCMDW SVGDVSYK-R GVFYLGAEFP TKEG------ |ADH's
SNINWAKGID EN------GR PIYDDANRPG DPAKSEDGKK GKSVYATPGF LGGKNWNPIA YSPDT-RLFY VPSNEWGMDI WNEPVNYK-K GAAYLGAGFT IKPT------ |
EKITWATGLD EN------GR PKFVADNRPG DPKDSKDGKQ GKSVFAVPSF LGGKNQQPMA YSPDT-KLFY VPSNEWGMDI HNEPVTYK-K GAAYLGAGFT IKPI------ |___
                                                   *    *                                          *
```

```
-PKGDRQNYE GLGQIKAYNA ITGDYKWEKM ERFAVWGGTM ATAGDLVFYG TLDGYLKARD SDTGDLLWKF KIPSGAIGYP MTY-THKGTQ YVAIYYGVGG WPGVGLVFDL |---
-PKGDRQNYE GLGQIKAYNA ITGKFKWEKM EKFAVWGGTM STAGDLVFYG TLDGFIKARN TDTGELLWKF KLPSGSIGYP ITY-THKGIQ YVAINYGVGG WPGVGLVFDL |
-PKGDRQNAE GLGQIKAYNA ITGKFKWDKM ERFAVWGGTL ATAGNVVFYG TLDGFIKARN SDTGELLWKF KLPSGAIGYP ITY-TRKGVQ YIAIYYGVGG WPAVGLVFDL |MxaF
-PKGDRQNYL GLGQIKAYNA ITNTYKWEHM ERFSVWGGTL ATAGNLVFYG TLDGFLKARN SDTGELLWKH KLPSGVIGYP MTY-EHKGVQ YIAVMSGVGG WPGVGLVFDL |___
-PK----GDS HMGNFTAWDA KTGKIVWQNK EQFSVWAGAV ATAGNVVFYG TLEGYLKAVN ATTGEELYKF KTPSGIIGNV ITY-EHAGKQ FVAVLSGVGG WAGIGLAAGL |
-PK----GDT HLGNFTAWDA KAGKIIWQKK EQFSVWSGAL ATAGNVVFYG TLEGYLKAVD AKTGDELFKY KTPSGIIGNV MTY-EHNGKQ YVAVLSGVGG WAGIGLAAGL |
-PK----GDT NLGNFTAWDA KAGKIVWQKK EPFSVWSGAL ATAGNVVFYG TLEGYLKAVD ARTGDELFKY KTPSGIIGNV ITY-EHKGKQ YVAVLSGVGG WAGIGLAAGL |
-PK----GES NMGNFIAWDA KVGKIVWSNK EQFSVWSGAL ATAGNVVFYG TLEGYLKAVD AKTGEELYKF KTPSGIIGNV MTY-EHKGKQ FVAILSGVGG WAGIGLAAGL |
-PG----GHG GMGNFIAWDN ISGKIKWSNP EQFSVWSGAL ATAGDVVFYG TLEGYLKAVD SKTGKELYKF KTASGVIGNV MTY-THKGKQ YVGVLSGVGG WAGIGLAAGL |
-PG----SHG GMGNFIAWDN LQGKIKWSNP EQFSAWGGAL ATAGDVVFYG TLEGFLKAVD SKTGKELYKF KTPSGIIGNV MTY-EHKGKQ HVAVLSGVGG WAGIGLAAGL |
-PN----SHG GMGNFIAWDN TTGEIKWSVP EQFSVWSGAL ATAGDVVFYG TLEGYLKAVD AQTGEELYKF KTPSGIIGNV MTY-EHGGKQ YVGILSGVGG WAGIGLAAGL |
-KG-----ET NMGALIGWDA ASKRVVWSDP EPFAVASGVL ATAGGLVFYG TLEGYLKAVD ARSGKELYRF KTPSGIIGDV TSF-ERNGRQ YIAVLSGAGG WSGVGLTAGL |XoxF5
-KG-----AA DMGGLTAWDA DARREIWSIR ERFAVVSGAL ATAGGLVFYG TLEGYLKAVD AKSGKELYRF KTPSGIVGDV AAF-DNGGRE YIAVLSGVGG WAGAGLTAGL |
-PD----SQG ATGGLIAWDG ARGKIVWSKP ERFAVWSGVL TTAGGIAFYG TLEGYFKAVD QKDGTELFKF KTPSGIVGNP MTY-RHAGKQ YVAVFSGVGG WAGIGLAAGL |
-PD----SHG GMGNFIAWDG VKGKIVWSKP EQFSVWSGAL TTAGGVAFYG TLEGYFKAVD QKDGKELFKF KTPSGIIGNA MTY-SHGGKQ YVAVFSGVGG WAGIGLAAGL |
-PE----GSD RTGAFIAWDG AKGNIAWTIP EAFSVWSGAL ATAGNVVFYG TLEGYLKAVD ARDGHELYRF KTPSGIVGNV TTY-QHGGRQ YVAVLSGIGG WAGIGLAAGL |
-PE----GSD RTGAFIAWDG AKGTIAWTIP EPFSVWSGAL ATAGNVVFYG TLEGYLKAVD ARDGHELYRF KTPSGIVGNV TTY-QHDGRQ YVAVLSGIGG WAGIGLAAGL |
GEK-E--SSN HMGSFTAWDP TTGTIAWQFD EPFSLWSGMV STAGDIVIYG TLEGYLKVRD AKTGEELYRF KTPSGIIGNV STW-TYNGKQ YIGVLSGIGG WAGVGMAAGL |
GKQ-D--GST NLGQFTAYDA TTGKIKWSNK ETFSVWSGSV ATAGGVVFYG TLEGYLKAVD AETGKELYRF KTPSGIIGNV NTW-SYKGKQ YVGVLSGIGG WAGIGIAAGL |
GKK-D--GST NLGQFTAYDA TTGKIKWSNK ETFSVWSGSV ATAGGVVFYG TLEGYLKAVD AETGKELYRF KTPSGIIGNV NTW-SYKGKQ YVGVLSGIGG WAGIGIAAGL |___
-PD------G VMGGFMAWDG LKGKSAWYNK EKFSAWGGAL VTSSDIVFYG TLDRWFKAVD AQTGKELWKF QVGSGVVGNA ITY-SHKGKQ YVGVLSGIGG WAGVAMNLGL |
-PD------G VTGGFMAWNG LTGKSAWYNK EPYSIWSGAM VTGADLVFYG TLDRWFKAVD AKSGKSLWQF QVGSGVIGNP FTY-SNKGKQ YVGVMSGIGG WAGIAINLGL |XoxF4
-PD------G VMGGFAAYDP MTNKAKWYNK EKFSAWGGAL ATASDLVFYG TLDRWFKALD AQTGKELWKF QVGSGVIGNA FTY-SHKGKQ YVGVLSGIGG WAGVAMNLGL |___
-PG------G HRGVYTAWDV ARERAAWSIK EEFPVWSGTL VTAGDITFYG TMDGWFKAVN ARTGEVVWQF KTGSGIIGQP TTYRGPDGRQ HVAILSGVGG WPGVVVSADL |
-EG------E NRGEYAAWDI LNGKKVWAIQ ERFPVWSGTV VTAGDVAFYG TMDGWFKAIN ANTGELLWQF KTGSGIIGQP VTYLGPDGKQ YVAVLSGVGG WSGAIVAGGI |XoxF3
-PG------G NRGEYAAWDI LKGKKVWAIK ERFPVWSGTV VTAGDIAFYG TMEGWFKAVN ANTGELLWQF KTGSGIISQP VTYRGPDGKQ YVAVLSGVGG WSGAIVAGGL |___
-EG----KDK YYGEFIAWDP VKGRRVWEIQ ERFPVWSGAL ATAGGLVFYG TLDGWFKAVD ARTGVLLWQQ KLGSGIVGNP IAFLGPDNKE YVAVYAGVGG WFGLPAAVNL |
-EG----RDK YYGEFIAWDP VKGRRVWEIQ ERFPVWSGAL ATAGGLVFYG TLDGWLKAVD ARTGVLLWQQ KLGSGIVGNP IAFLGPDNKE YIAVYSGVGG WFGLPAAVSL |
-EG----KDK YYGEFIAWDP VKGRRAWEIQ ERFPVWSGAL ATAGGLVFYG TLDGWFKAVD ARTGVLLWQQ KLGSGIVGTP IAFLGPDNKE YVAVYSGVGG WFGLPVAVDL |
-EG----QDK YYGEFIAWDP VKARRAWEIQ ERFPVCSGAL ATSGGLVFYG TLDGWFKAVD ARTGVVLWQQ KLGSGIVGSP IAFLGPDNKE YVAVYSGIGG WFGLPVALDL |
-EG----RDK YYGEFIAWDP VKGRRAWELQ ERFPVCSGAL ATAGGLVFYG TLDGWFKAVD ARTGVLLWQQ KLGSGIVGSP IAFLGPDNKE YVAVYSGVGG WFGLPAALDL |
-EG----KDD YYGAFICYDA LKGKRVWEIH EHFPVWSGPV VTAGGLAFYG TMDGWFKAVD IKTGKVLWQQ KLGSGIIGNP ITYLGPDKKQ YVAVYSGVGG WFGIAVAQNL |XoxF2
-EG----KDD YYGAFICYDA LKGKRVWEIH EHFPVWSGPV VTAGGLAFYG TMDGWFKAVD IKTGKVLWQQ KLGSGIIGNP ITFLGPDKKQ YVAVYSGVGG WFGIAVAQNL |
-EG----KDD YYGAFICYDA LKGKRVWEIH EHFPVWSGPV VTAGGLAFYG TMDGWFKAVD IKTGKVLWQQ KLGSGIIGNP ITFLGPDKKQ YVAVYSGVGG WFGIAVAQNL |
-EG----KDD YWGEFIAWDP VKGRKVWGIK EKFPVWSGVL TTAGGVVFYG TMDGWFKAVD AKTGKILWQH KVGSGVIGNP MTFLGPDKKQ YVAVYTGVGG WFGLPVAQGL |
-EG----KDD YYGAFICYDA LKGKRVWEIH EHFPVWSGPV VTAGGLAFYG TMDGWFKAVD IKTGKVLWQQ KLGSGIIGNP ITFLGPDKKQ YVAVYSGVGG WFGIAVAQNL |
-EG----KDD YYGAFICYDA LKGKRVWEIH EHFPVWSGPV VTAGGLAFYG TMDGWFKAVD IKTGKVLWQQ KLGSGIIGNP ITFLGPDKKQ YVAVYSGVGG WFGIAVAQNL |
-EG----KDD YYGAFICYDA LKGKRVWEIH EHFPVWSGPV VTAGGLAFYG TMDGWFKAVD IKTGKVLWQQ KLGSGIIGNP ITFLGPDKKQ YVAVYSGVGG WFGIAVAQNL |___
-PG------- VMGKLKKFNV LTGKTEWEIP DAYPNWSGTL NTDGGLVFYG SLNGDFRAVD RATGKVLWQR KLGSGVIGNP IAY-KIKGHE YVSVLSGIGG WIGLPAVAGL |
-PG------- VTGKLKKFDV LTGKTAWEVP DAYPNWSGTL NTAGGLVFYG SLNGDFRAVD RDNGKVLWQR KLGSGIIGNP IAY-KIKGHE YISVFAGIGG WIGLPAVAGL |
-PG------- VTGKIKKYDV LTGQTDWEIP DQYPNWGGTL NTSGGLMFYG SLGGDFRAVD RNTGKVLWDR KLASGIIGNP ITY-KIKDKQ YVSILSGIGG WIGVPVTAGL |
-PG------- TTGKIKKFDV LTGTADWEIP DPYPNWGGTM VTDGGLVFYG SLGGDFRAVD RKSGKILWHR KLASGIIGNP ITY-KVKGKQ YVSILAGIGG WIGVPVTAGL |
-PG------- VMGRFKAFDV ITGKARWDIP ERFPTWSGAL VTDGGLAFYG TLDGWFKAVD RKTGKVLWQQ KLGSGIIGNP ISY-EVGGKQ YISVLSGIGG WIGLPVTAGL |XoxF1
-PG------- VLGRFKAFDV VTGKARWDIP ERFPTWSGAL VTDGGLAFYG TLDGWFRAVD RKTGKILWQQ KLNSGIIGNP ISY-EVNGKQ YVSILTGIGG WIGLPVTAGL |
-PG------- VMGRFKAFDV ITGKARWDIP ERFPTWSGAL VTDGGLAFYG TLDGWFRAVD RKTGKVLWQQ KLGSGIIGNP ISF-EVGGKQ YISILTGIGG WIGLPVTAGL |
-PG------- VMGRFKAFDV ITGKARWDIP ERFPTWSGAL VTDGGLAFYG TLDGWFRAVD RNSGKVLWQQ KLGSGIIGNP ISY-EVGGKQ YISILTGIGG WIGLPVTAGL |
-PG------- VLGRFKAFDV ITGKARWDIP ERFPTWAGAL VTDGGLAFYG TLDGWFRAVD RKTGKVLWQQ KLGSGIIGNP ITF-EVGGKQ YVSILTGIGG WIGLPVAAGL |___
-PG------G FLGELIAWDP VNNKKVWSIQ EDLPFNGGTL STAGGLVFSG NLHGDFRAID AKSGKILWKK NLGSGIGAGP VTY-SVDGKQ YVAVV--VGR TASIPAF--- |
-PG------G FLGELVAWDP VANKKVWSIK EDLPFNGGTL STAGGLVFSG NLHGDFRAVD AKTGNILWKK NLGSGIGAGP VTY-SVDGKQ YVAVV--VGR TASIPAF--- |ADH’s
-------FED HIGSLKAIDP ATKKVVWEYK NKAPLWAGVL TTAGNLVFTG TPEGFLKAFD AKTGEEVWKF QVGTGVVASP ITW-EQDGEQ WIGVAAGWGG --AVPLWGGE |
-------FED HIGALKAVDP ATGKIVWQIN NKAPLWGGVL TTGGGLVVYG TPEGDLKVVD AKDGKELWHF NTGSGIVAPP ITW-EQDGEQ MISVISGWGG --AVPLWGGE |___
            *            *          *    *      *              *          *                      *
```

```
ADPTA----- GLGAVGAFKK LA--NYTQMG GGVVVFSLDG KGPYDDPNVG EWKSAAK |---
QDPTA----- GLGAVGAFKK LA--HYTQMG GGTMVFSLDG KGPYDDVNLG EFASSK- |
QDPTA----- GLGAVGAFKQ LA--NYTQQG GGVLVFSLNG KGPYDDPNVG EYQSAN- |MxaF
QDPTA----- GLGAVGAFKN LQ--RYTQMG GSLEVFSLDG KNPYDDVNVG EWTKG-- |___
TNPTA----- GLGAVGGYSS LA--NYTALG GQLTVFALPE Q--------- ------- |
TKGTE----- GLGAVGGYAS LA--NYTALG GQLTVFALPN ---------- ------- |
SKGTE----- GLGAVGGYAS LA--NYTALG GQLTVFALPG K--------- ------- |
TDPNA----- GLGAVGGYAA LS--NYTALG GQLTVFALHG K--------- ------- |
TDPNA----- GLGAVGGYAA LS--QYTNLG GQLTVFALPN ---------- ------- |
TDPNA----- GLGAVGGYAA LS--SYTNLG GQLTVFSLPN N--------- ------- |
TNPND----- GLGAVGGYAS LS--QYTELG GQLTVFELPG ---------- ------- |
THSDE----- GANSPGVYAA LA--NYTALG GQLTVFALK- ---------- ------- |XoxF5
TQTPE----- GANSPEVYAA LA--QYTALG GQLTVFALP- ---------- ------- |
TNPDD----- GSGAVGGFAS LS--GYTSLG GSLTVFALP- ---------- ------- |
TNPTD----- GLGAVGGYAG LK--QYTNLG GSLTVFALP- ---------- ------- |
TDPAD----- GSGAVGGYAA LS--QYTAPG GQLTVFALPE ---------- ------- |
TDPAD----- GSGAVGGYAA LS--QYTAPG GQLTVFALPE ---------- ------- |
EGDTE----- GLGAVGAYKG LS--SHTKLG GVFTVFALP- ---------- ------- |
DDGSDSTNTE GLGAVGAYRS LS--SFTKLG GTFTVFALPG K--------- ------- |
DDGSDSTNTE GLGAVGAYRS LS--SFTKLG GTFTVFALPG K--------- ------- |___
TNETD----- GLGAAGGYKE LTKWNAAPGG GAMNVFSL-- ---------- ------- |
TEDSD----- GAGAAGAYRE LTKWNAAPGG GAVSAFSL-- ---------- ------- |XoxF4
ENETD----- ALGAAGGYKE LTKYNAAPGG GALNVFSL-- ---------- ------- |___
DPRDATG--- ALGFLNAMRD LK--KVTTKG GMLYDFVLP- ---------- ------- |
DKRDGTA--- ALGFVNAMKD LP--EVTTKG GELYVFALP- ---------- ------- |XoxF3
NKMDGTA--- ALGFVNAMKD LP--DVTTKG GMLYVFALP- ---------- ------- |___
PKDDPRA--- AMGANGAADD SGLPQSTTPG GELVVFGLD- ---------- ------- |
PKDDPRA--- AMGANGAADN SGLPQSTTPG GELVVFGLD- ---------- ------- |
PKSDPRA--- AMGADGAAYA SHLPESTTPG GELVVFGLD- ---------- ------- |
PKGDPRA--- AMGAAGAAYD SGLPESTTPG GELVVFGLE- ---------- ------- |
PRGDRQG--- AMGASGAAYD SGLPGSTTPG GELVVFGLE- ---------- ------- |
PPDDPYA--- GLGAVGVAYQ AGLPKATTIG GELYVFSLE- ---------- ------- |XoxF2
PPDDPYA--- GLGAVGVAYQ AGLPKATTIG GELYVFSLE- ---------- ------- |
PPDDPYA--- GLGAVGVAYQ AGLPKATTIG GELYVFSLE- ---------- ------- |
PPDDPFG--- ALGAIGAAYQ SGLDKATSVG GELVVFALP- ---------- ------- |
PPDDPYA--- GLGAVGVAYQ AGLPKATTVG GELYVFALE- ---------- ------- |
PPDDPYA--- GLGAVGVAYQ AGLPKATTIG GELYVFSLE- ---------- ------- |
PPDDPYA--- GLGAVGVAYQ AGLPKATTIG GELYVFSLE- ---------- ------- |___
DLDDKFG--- AIGSTALTKV GGF-NKIPQG GTLYTFRVPD KAASTQ---- ------- |
DLEDKFG--- AIGSTALTKV IGL-NKIPQG GALYTFRVPD KEATAH---- ------- |
DLNDKFG--- AIGATAMTKA ANL-DKIPQA GTLFTFRVME ---------- ------- |
DLNDKFG--- AIGATAMTKA ANL-DKIPQA GTLFTFRVFE ---------- ------- |
DPADPYG--- ALGVSGMAAE NGF-YNIPMG GTLYTFCVQ- ---------- ------- |XoxF1
DPSDPYG--- ALGCSGMAAE NGF-YNIPMG GTVYTFCVQ- ---------- ------- |
DPADPYG--- ALGVAGMAGE NGF-YNIPMG GTLYTFCVQ- ---------- ------- |
DPADPYG--- ALGVAGMAGE NGF-YNVPMG GTLYTFCVQ- ---------- ------- |
DPSDPYG--- ALGDSGLAAD NGF-YNIPMG GTLYTFCVQ- ---------- ------- |___
---------- -LGEIGK--- -KMTNAAPEG GALFVFAVQ- ---------- ------- |
---------- -LGEIGK--- -KMTNAAPEG GALFVFAVQ- ---------- ------- |ADH's
VAKTT----- ---------- ----AGINQG GSFWAFRLPK ALASR----- ------- |
VAKTV----- ---------- ----QHLNQG GMVWTFKIPK KTASN----- ------- |___
                                 *    *
```
