# Supplementary material for: Neodymium as Metal Cofactor for Biological Methanol Oxidation: Structure and Kinetics of an XoxF1-Type Methanol Dehydrogenase
Source: mBio. 2021 Sep 21;12(5):e01708-21. doi: 10.1128/mBio.01708-21 (PMC8546591; doi:10.1128/mBio.01708-21)
Supplement: FIG S4 [file mbio.01708-21-sf004.pdf]

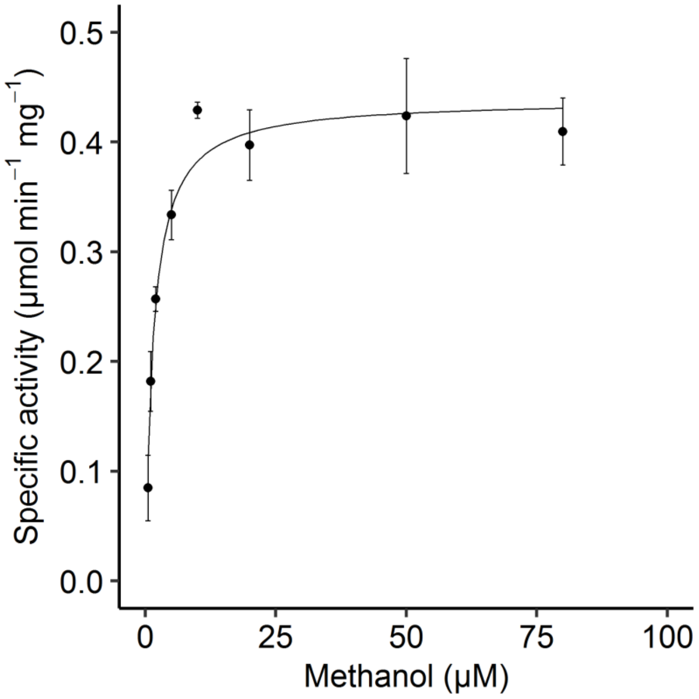

Figure S4: Specific enzyme activity of methanol oxidation by Nd-XoxF1 measured spectrophotometrically in a 4 mL cuvette.
