# Supplementary figures and images for: Neodymium as Metal Cofactor for Biological Methanol Oxidation: Structure and Kinetics of an XoxF1-Type Methanol Dehydrogenase
Source: mBio. 2021 Sep 21;12(5):e01708-21. doi: 10.1128/mBio.01708-21 (PMC8546591; doi:10.1128/mBio.01708-21)

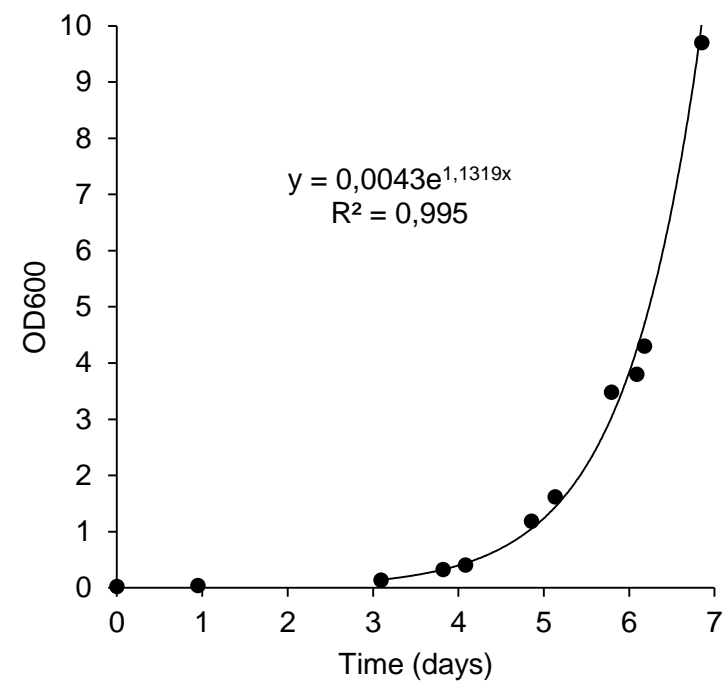

**Figure S1:** Growth curve of *Methyloacidimicrobium thermophilum* AP8 in a batch chemostat.

Supplement: FIG S1 [file mbio.01708-21-sf001.pdf]
