# Supplementary material for: Neodymium as Metal Cofactor for Biological Methanol Oxidation: Structure and Kinetics of an XoxF1-Type Methanol Dehydrogenase
Source: mBio. 2021 Sep 21;12(5):e01708-21. doi: 10.1128/mBio.01708-21 (PMC8546591; doi:10.1128/mBio.01708-21)
Supplement: TABLE S1 [file mbio.01708-21-st001.pdf]

Table S1: Diffraction data and crystallographic model statistics. Values for the highest resolution shell are given in parentheses.

	**PDB 7O6Z**
**Data collection**	
Space group	$P2_1$
Unit cell dimensions	
a, b, c [Å]	89.5, 70.0, 96.5
α, β, γ [°]	90, 91.7, 90
Wavelength [Å]	0.99985
Resolution range [Å]	48.6-2.3 (2.4-2.3)
No. measurements	103,564 (7,698)
Unique reflections	51,250 (3,810)
Completeness [%]	94.8 (96.1)
Redundancy	2.0 (2.0)
$I/\sigma(I)$	8.4 (1.2)
R_{merge}	0.065 (0.787)
R_{meas}	0.087 (1.048)
$CC_{1/2}$	99.7 (61.8)
**Refinement**	
Resolution range in refinement [Å]	48.6-2.3
Number of reflections in refinement	51,183
R_{work}/ R_{free}	0.1952 / 0.2529
Protein residues / no. of atoms	1176 / 9546
Ligands / no. of atoms	4 / 30
Overall B-factor [Å^2]	53.57
Rmsd from ideal geometry	
bonds [Å]	0.005
angles [°]	1.14
%-age residues in areas of Ramachandran plot	
Most favored	93.43
Additionally allowed	6.23
Outliers	0.34
